# A genome-wide scan for signatures of directional selection in domesticated pigs

**DOI:** 10.1186/s12864-015-1330-x

**Published:** 2015-02-25

**Authors:** Sunjin Moon, Tae-Hun Kim, Kyung-Tai Lee, Woori Kwak, Taeheon Lee, Si-Woo Lee, Myung-Jick Kim, Kyuho Cho, Namshin Kim, Won-Hyong Chung, Samsun Sung, Taesung Park, Seoae Cho, Martien AM Groenen, Rasmus Nielsen, Yuseob Kim, Heebal Kim

**Affiliations:** Department of Agricultural biotechnology, Seoul National University, Seoul, 151-921 Republic of Korea; Department of Life Science and Division of EcoScience, Ewha Womans University, Seoul, 120-750 Republic of Korea; Animal Genomics and Bioinformatics Division, National Institute of Animal Science, Rural Development Administration, Suwon, 441-706 Republic of Korea; Interdisciplinary Program in Bioinformatics, Seoul National University, Seoul, 151-747 Republic of Korea; C&K Genomics, Seoul National University Research Park, Seoul, 151-919 Republic of Korea; Animal Genetic Resources Station, National Institute of Animal Science, Rural Development Administration, Suwon, 441-706 Republic of Korea; Swine Science Division, National Institute of Animal Science, Rural Development Administration, Suwon, 441-706 Republic of Korea; Korean Bioinformation Center, Korea Research Institute of Bioscience and Biotechnology, Daejeon, 305-806 Republic of Korea; Department of Statistics, Seoul National University, Seoul, Republic of Korea; Animal Breeding and Genomics Centre, Wageningen University, De Elst 1, 6708 WD Wageningen, The Netherlands; Department of Integrative Biology and Department of Statistics, University of California Berkeley, Berkeley, CA 94820 USA; Current address: Department of Genome Sciences, University of Washington, Seattle, WA USA

**Keywords:** Pig, Domestication, Selective sweep, Directional selection, Quantitative traits

## Abstract

**Background:**

Animal domestication involved drastic phenotypic changes driven by strong artificial selection and also resulted in new populations of breeds, established by humans. This study aims to identify genes that show evidence of recent artificial selection during pig domestication.

**Results:**

Whole-genome resequencing of 30 individual pigs from domesticated breeds, Landrace and Yorkshire, and 10 Asian wild boars at ~16-fold coverage was performed resulting in over 4.3 million SNPs for 19,990 genes. We constructed a comprehensive genome map of directional selection by detecting selective sweeps using an *F*_ST_-based approach that detects directional selection in lineages leading to the domesticated breeds and using a haplotype-based test that detects ongoing selective sweeps within the breeds. We show that candidate genes under selection are significantly enriched for loci implicated in quantitative traits important to pig reproduction and production. The candidate gene with the strongest signals of directional selection belongs to group III of the metabolomics glutamate receptors, known to affect brain functions associated with eating behavior, suggesting that loci under strong selection include loci involved in behaviorial traits in domesticated pigs including tameness.

**Conclusions:**

We show that a significant proportion of selection signatures coincide with loci that were previously inferred to affect phenotypic variation in pigs. We further identify functional enrichment related to behavior, such as signal transduction and neuronal activities, for those targets of selection during domestication in pigs.

**Electronic supplementary material:**

The online version of this article (doi:10.1186/s12864-015-1330-x) contains supplementary material, which is available to authorized users.

## Background

Identification of genes under selection is a major goal in the study of domestication in animals [1-4] and plants [5]. The process of domestication, accompanied by selection on traits related to yield, morphology, fertility and survival during captive breeding, is believed to have dramatically affected the frequency of alleles segregating among domesticated breeds [6,7]. Mutations conferring new favorable phenotypes will be subject to a ‘selective sweep’, a rapid increase in allele frequency by artificial selection. Breeds affected by such sweeps will harbor large genetic differences with other breeds and carry signatures of selection in the genomic regions involved [8-12].

Recent genome-wide scans in diverse breeds aimed to uncover the genetic basis for phenotypic variation in pigs [3,4] showed that selection mapping approaches can detect comprehensive signatures of intense artificial selection that have led to the formation of well-defined breeds, suggesting that domestic animals can serve as models for deciphering complex phenotype-genotype association through selection mapping [3]. Previous studies suggested that European and Asian pigs were derived from multiple independent domestication events [13-15], notably from European and Asian subspecies of wild boars that are estimated to have split about ~1 million years ago [7], followed by the occurrence of introgression of Asian pigs into some European breeds during the Neolithic [14] and 18^th^-19^th^ centuries [16-19]. Although the demographic history of pig domestication is highly complicated, recent studies have identified candidate genes with distinct patterns of differentiation underlying the phenotypic diversity of breeds [2,4,20], suggesting that the breed formation results in fixation of genetically differentiated gene pools within the regions under the artificial selection exercised by breeders.

To access a comprehensive analysis of genetic variations underlying domestication traits in the well-established pig breeds (i.e. Landrace and Yorkshire), we focused on investigating highly distinct patterns in genes under the artificial selection by two different approaches: an *F*_ST_-based approach that detects directional diversifying selection [8] and a haplotype-based test that detects very recent selective sweeps within breeds [21]. The *F*_ST_-based statistic detects strong shifts in allele frequencies to a fixed difference between local populations. The signatures detected here are likely to capture directional selection that occurred during or shortly after the establishment of the respective breeds [22,23]. And, the haplotype-based statistic detects a rapid rise of a selected allele to an intermediate frequency during which the long-range of haplotype association is not eliminated by recombination [24]. These signatures are likely to capture positive selection for variants that occurred after the separation between the European and Asian pigs, and where the alleles have not reached fixation in European breeds. Our previous study on the phylogenetic diversity of the Asian wild boar and European breeds showed that the Korean wild boars can serve as a distinctive outgroup to differentiate European breed-specific genetic variations during domestication [25]. Growing evidence suggests that the sweeps and directional selection are associated with quantitative traits in domesticated animals, like pigs [3], chickens [26], cattle [27], and dogs [28].

In this study, we applied both methods to whole genomes of two major domesticated breeds, Landrace and Yorkshire, using Asian wild boars as an outgroup. Distinct patterns of selection signatures were found at loci that may contribute to domestication phenotypes, including behavior. We further annotated candidates of artificial selection with our studies and those in previous QTL mapping studies. We suggest that signatures of distinct patterns of genetic variation detected here are valuable resources to integrate QTL information and genetic candidates into our understanding of the phenotypic variation in pig domestication.

## Results

### Population structure

**Figure 1:**
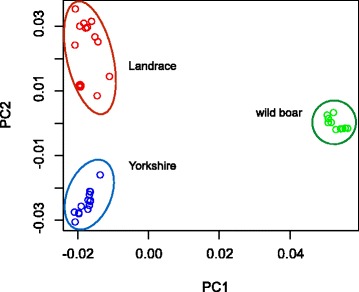
**Population structure of European domesticated pigs and Asian wild boar.** The fraction of the variance explained is 57.5% for eigenvector 1 and 26.6% for eigenvector 2.

We resequenced the whole genomes of Yorkshire (*n* = 16), Landrace (*n* = 14), and Asian wild boar (*n* = 10) at an average depth (± s.e) of 16.1 ± 0.8, 14.6 ± 0.5, and 15.4 ± 0.4, respectively. First, we examined the genetic diversity (π) in the genomes. π was significantly lower (Wilcoxon-test, *p*-value < 10^−16^) for Yorkshire and Landrace (0.0029 ± 3.0 × 10^−6^ and 0.0028 ± 3.0 × 10^−6^, respectively) than that of wild boar (0.0036 ± 3.0 × 10^−6^), reflecting a possible genetic bottleneck or founder effect in domesticated breeds. Next, to examine the population structure among breeds, we analyzed SNP genotype frequencies with ADMIXTURE [29] and performed a multidimensional scaling (MDS) analysis using PLINK [30]. The MDS analysis indicates the partitioning between European pigs from Asian wild boars on the first two PC axes. PC1 depicts the Asian wild boars versus European pig axis, and PC2 represented the genetic difference between European breeds (Figure 1). ADMIXTURE recapitulated the partitioning of the Asian wild boar and the European domesticated lineages for varying numbers of ancestral populations (*K*) (Additional file 1: Figure S1). Thus, genome-wide scans for signatures of diversifying selection relative to Asian boars would detect the loci of directional selection in the European domesticated breeds.

### Low haplotype diversity in domesticated pigs

**Figure 2:**
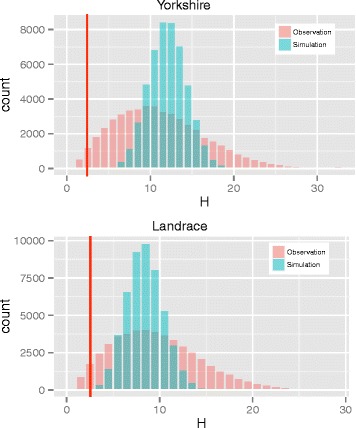
**Distribution of the number of distinct haplotypes (** ***H*** **).** *H* is measured by counting the number of unique haplotypes for bins with 30 variable sites within each breed. A neutral simulation without recombination under the inferred wild boar/Yorkshire/Landrance demography was carried out to compare the distribution of the observed *H* and that of simulated *H*. Red vertical line depicts a part of observed H showing complete linkage disequilibrium with only a few distinct haplotypes in the pig genome.

Upon visual inspection of phased sequence alignments, we noticed that Yorkshire and Landrace genomes are enriched for large clusters of SNPs in strong linkage disequilibrium (LD). Such haplotype structure is captured by the distribution of the number of distinct haplotypes, *H*, in a sliding window of 30 consecutive SNPs within each population. Figure 2 shows that a large number of windows exhibit complete linkage disequilibrium with only a few distinct haplotypes (*H* = 2 ~ 4). We tested if simple demographic structure can explain such patterns of haplotypes, by constructing a simple demographic model for the three populations resulting from two population splits, the first at the foundation of an ancestral domesticated lineage and the second at the formation of the two breeds (details in Methods). We assume a constant migration rate between breeds after the populations split. After obtaining the best-fit parameters of the model, we generated data by neutral coalescent simulation under the inferred demography. We find that the extremely low haplotype diversity observed in Yorkshire and Landrace is not generated by the simulation even when a recombination rate of zero is used (Figure 2).

To account for this low haplotype diversity in domestic breeds, we examined if our data set violated the assumption of random sampling of unrelated individuals, by testing potential family structure in our samples that may arise due to modern breeding practices. We used RelateAdmix [31] and confirmed that our sampled individuals are indeed unrelated (individuals are most likely separated for more than first-cousin relationships (4 ≥ generation); Additional file 1: Figure S1). A plausible explanation might be recent admixture that occurred during the complicated breeding history of these breeds, in which crosses are made between genetically divergent breeds that also experienced severe genetic bottlenecks. Under this perspective, a complex demographic model, incorporating multiple independent derivations of domesticated populations from wild boar followed by inbreeding and recent admixture among them, is likely needed to account for the observed low haplotype diversity [7,17,32]. Thus, instead of using a model-based approach, which involves inferring complex demographic parameters for domesticated pigs to approximate the null distribution, we followed an outlier approach to identify candidate genes under selection by taking 99^th^ percentile of the empirical distribution. This approach has been shown to be useful in studying such samples as domesticated populations [2,4,33].

### Mapping selective sweep in the domesticated pig breeds

We scanned the signatures of selection that is predicted to alter allele frequencies and haplotype structure within domesticated population. First, genome-wide *PBS* was calculated for a sliding window of 200 consecutive SNPs in Landrace and Yorkshire populations using wild boar as an out-group population, for detecting lineage specific reduction of allele frequencies. There is a negative relationship between *PBS* and the nucleotide diversity of domesticated lineages relative to wild boar (Additional file 2: Figure S2), indicating, as expected, that the signal of selection is most pronounced where nucleotide diversity is reduced in the domesticated lineage. Next, genome-wide integrated haplotype score (*iHS*) was calculated to detect long-range haplotype structure associated with directional selection [24]. Because the *iHS* has its maximal power when selected alleles segregate at intermediate frequency, we limited eligible SNPs to those with MAF > 0.2 in each breed. High *iHS* values are evidence for ongoing directional selection that rapidly increase the selected allele frequency along with longer haplotype background of the selected alleles than that of the alternative allele. (Additional file 3: Figure S3).

**Figure 3:**
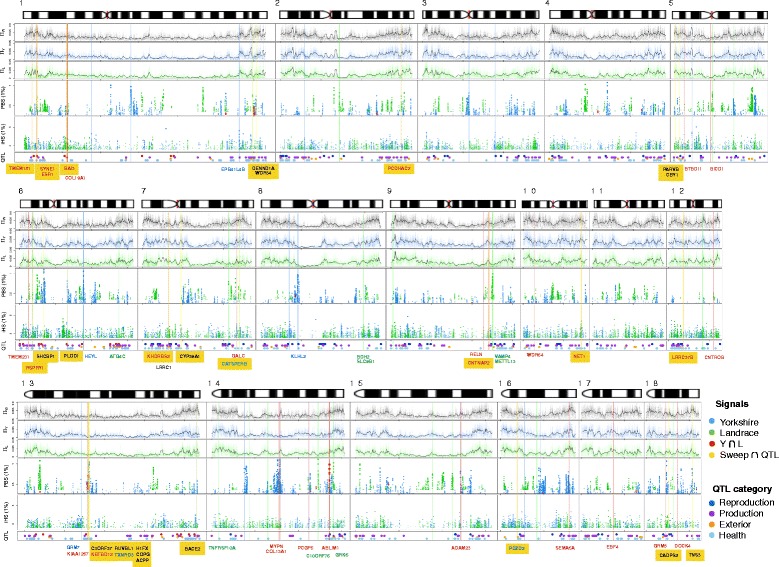
**High-resolution map of artificial selection in domesticated pig.** Red color depicts signatures of selection showing strong signals (top 1%) detected in both Yorkshire (green) and Landrace (blue). Part of common signatures is detected only by PBS, indicated by red spots, otherwise, detected only by *iHS*. Yellow color depicts signatures of selection showing strong signals in Yorkshire and/or Landrace that overlapped with QTL. Of those signals, genes within the top 50 in Yorkshire and Landrace (red) are annotated below the vertical lines. Of those signals, genes with top five Yorkshire-specific (blue) and top five Landrace-specific signals are shown below corresponding vertical lines.

As our primary goal is to identify putative candidate genes involved in pig domestication (Figure 3), we only considered bins/sites yielding large values of *PBS*/*iHS* located in the genic regions of the genome for further analyses, where a genic region is defined as one of the 19,990 reference genes in the reference pig genome. By sorting genes by the strength of signal mapped to them, we identified the top 200 candidate genes that were deemed as outlier values at 99^th^ percentile of all genes (Additional files 4,5,6, and 7: Figure S4, S5, S6 and S7, details in methods) for each combination of breed and detection method. Strong between- and/or within-population differentiation of haplotypes indicative of local or partial selective sweeps are observed in the alignment of variable sites in candidate genes harboring the strongest signals (e.g. Additional file 8: Figure S8). The clarity of such patterns is expected to diminish as the magnitude of selection signal decreases. However, even for candidates ranked at the bottom of the list of each test, we could still observe the qualitative patterns of directional selection, e.g., reduced diversity and/or increased haplotype homozygosity (Additional file 9: Figure S9). Thirty-one genes are found in common between the lists of candidates detected by *iHS* in Yorkshire and Landrace (Additional file 10: Figure S10), which is a statistically significant overlap (*p* < 10^−5^, when tested by bootstrapping with 100,000 replicates). However, such significant between-breed sharing is not observed among candidates detected by *PBS*, presumably because the method detects breed specific selection.

### Selective sweep mapping associated with quantitative traits loci in pig

For genes with putative signatures of directional selection, we investigated how many of them overlap with previously identified quantitative trait loci (Figure 3). We sought to annotate their potential roles in the process of domestication-related phenotypes rather than their broad functional terms in GO categories. For various trait categories, we identified QTL candidate genes as those located within the QTL-intervals on the genetic maps archived in the AnimalQTL database [34]. The sum of QTL intervals for a given trait sub-category covers 5 ~ 8% of the reference genes. In total, 4055 (20.3%) genes were associated with one or more quantitative traits. By using PBS (Additional file 11: Figure S11A), 50 and 54 selection candidates identified in Yorkshire and Landrace, respectively, also overlap with QTL-candidate loci, which represent statistically significant overlaps (*p* = 0.039 and 0.0046, respectively). By using the *iHS* method (Additional file 11: Figure S11A), 55 and 58 candidate genes identified in Yorkshire and Landrace are also overlapping with QTL-candidate loci (*p* = 0.007 and 0.0001), respectively. We also observe a large proportion of overlap between selection candidates detected by both PBS and iHS methods and QTL candidates identified from previously published association studies (Additional file 11: Figure S11B): out of the total 399 selection candidate genes in Yorkshire, 104 (26.1%) genes are QTL-candidate genes (*p* = 3.50 × 10^−7^). Out of the total 398 selection candidate genes in Landrace, 111 (27.8%) genes are QTL-candidate genes (*p* = 1.72 × 10^−6^). The overlap remains significant when method-specific selection candidates and QTL candidates are examined (Additional file 11: Figure S11C; *p* = 0.00015 and 7.79 × 10^−8^ for *PBS* and *iHS*, respectively).

**Figure 4:**
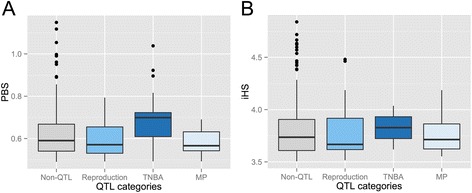
**Distribution of selection signals associated with reproduction.** Population-specific allele frequency change **(A)** and integrated haplotype score **(B)** for European breeds show similar patterns of selection at reproduction QTL and non-QTL. TNBA (Total Number of Born Alive) and MP (Mummified Pigs) entries are separated from the reproduction QTL category to distinguish potential effects of artificial selection on genes associated with specific measures of reproductive performance. Average PBS in TNBA is significantly higher (*p* < 0.05) compared to those in other traits.

When the QTLs are divided into four trait categories, selection candidate genes have a significant overlap with QTL candidates in the ‘*Reproduction’* and ‘*Exterior’* categories (Additional file 12: Figure S12). Using the PBS method, 31 genes (*p* = 0.00023) and 21 genes (*p* = 0.0043) detected in Yorkshire and Landrace, respectively, are ‘*Reproduction’* QTL candidate genes. However, we do not observe such enrichment of selection candidates by PBS or *iHS* in the ‘*Production’* QTLs. Using the *iHS* method, 17 genes (*p* = 0.00013) and 20 genes (*p* = 0.013) detected in Yorkshire and Landrace, respectively, are ‘*Exterior’* QTL candidate genes (Additional file 12: Figure S12). In total, 24 sub-categories of quantitative traits significantly (Bonferroni corrected *p* < 0.05) overlapped with putative candidate genes under strong artificial selection (Additional file 13: Table S1). The top five strong selection genes associated with QTLs in both Yorkshire and Landrace, in Yorkshire alone, and in Landrace alone are listed in Additional file 14, 15, 16: Tables S2, S3 and S4, respectively. In ‘*Reproduction’* categories, genes assigned to ‘*Total Number of Born Alive’* (Additional file 17: Table S5) are particularly interesting, as these are clustered on chromosome 13 (Figure 4). This cluster might reflect that these common candidate genes play a crucial role in the domestication-related phenotypes, and, thus are under strong artificial selection during pig domestication.

**Figure 5:**
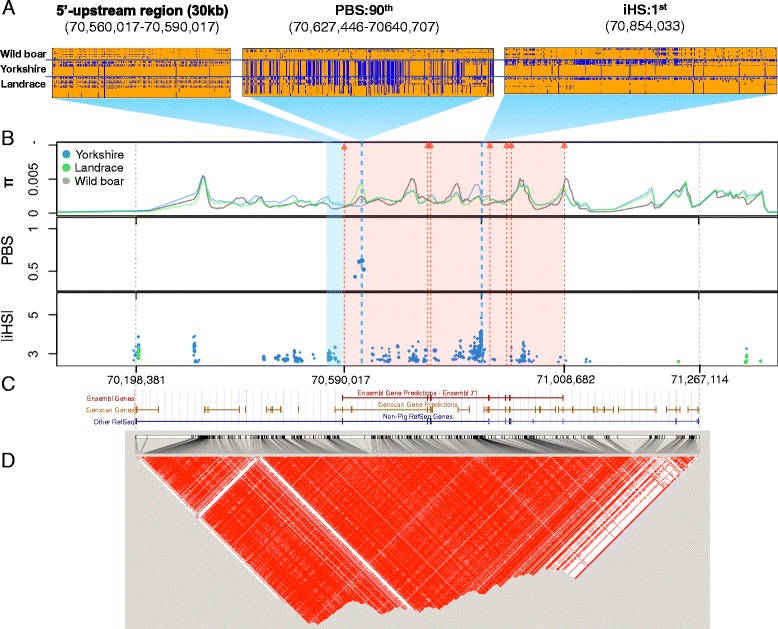
**Signatures of selection at GRM7 in the pig.** Structure of sequence variation around genes showing the strongest selection signal in Yorkshire **(A)**. Only variable sites are shown in the alignment of sequences for wild boar (W), Yorkshire (Y) and Landrace (L). The ancestral and derived alleles are colored in orange and blue, respectively. Variable sites located up to 15 kb up-stream and 15 kb down-stream from the focal bin/site (red dashed box/red vertical line) are shown. Test statistics shown are log_2_(*π*_T_/max(*π*_1_, *π*_2_)), π, *PBS* above the top 1% value (bins), and |*iHS*| above the top 1% value (sites) **(B)**. Red dashed arrows indicates the locations of exons of *GRM7*. The corresponding gene structure of *GRM7* **(C)**. Detailed linkage disequilibrium structure of *GRM7* dictated in HaploView. Increasing intensities of red represent higher *r* ^*2*^ values **(D)**.

It is quite possible that many genes subject to artificial selection during domestication could not be detected by QTL mapping because the phenotypes of many traits, e.g. immune/defense processes and behavior, cannot be easily scored or typed for QTL/association studies. In fact, a functional enrichment analysis shows that strong selection candidates involved with signal transduction (*p* = 5.1 × 10^−4^) and neuronal activities (*p* = 0.04). One of these genes exhibiting the strongest *iHS* in Yorkshire, *GRM7*, was not detected by QTL studies (Figure 5). There is prior evidences that *GRM7* impact specific brain function associated with spatial learning, memory, understanding of speech, and autism in humans [35]. Along with *GRM8*, which is also rated high in both in Landrace (*iHS*:19^th^) and Yorkshire (*iHS*:172^nd^) but does not overlap with QTLs, *GRM7* constitutes the group III metabotropic glutamate receptors (mGluRs), which inhibit neutrotransmitter release at the majority of excitatory synapses in the mammalian central nervous system [35]. It is to note that no nonsynonymous mutation in these genes was observed neither in Yorkshire nor Landrace, likely indicating that strong selection detected in domesticated breeds might act on the regulatory region of these genes.

## Discussion

To detect recent selective sweeps, we used two complementary methods (*PBS* and *iHS* tests). Both methods have power primarily to detect candidates of recent domestication events but of different types of selection. Both approaches are necessary in order to map the comprehensive footprint of selection in the genome and to construct a comprehensive selection map for the pig genome.

Both selective sweep mapping and QTL mapping have potential to detect genes under artificial selection during domestication. However, each approach has its own limit: the former may be affected by directional selection not related to domestication and does not inform us about the phenotype under selection. The latter may map loci of phenotypes that are not targets of domestication selection, for example phenotypic differences caused by transient (deleterious) mutations, and is unable to study phenotypes that cannot be clearly scored in a controlled environment. This study demonstrated the advantage of combining these two approaches and reveals a list of genes with clear contribution to domestication processes.

### Genetic variants underlying artificial selection during domestication

Putative signatures of selection can be considered as candidates for the development of domesticated pig breeds with well-defined traits over the past hundreds of years. A number of regions showing strong selection have been identified in previous studies [4,36,37].

A consequence of 300 years of artificial selection in Europe and the USA for enhancing faster growth under high-calorie uptake, 182 positively selected genes (PSGs) out of 7,917 orthologs were found by an increased ratio of nonsynonymous to synonymous substitutions shared in the Duroc genome and Tibetan wild boar compared to human, showing PSGs are enriched in the focal adhesion, muscle growth, and energy metabolism [36]. We found 14 genes identified in this study to overlap with those PSGs (Table 1), and five of them, including *ABLIM1*, *CXADR*, *INSR*, *RIMS1*, and SYNE1, to be related with regulation of developmental growth and anatomic structural development. For example, *CXADR,* identified in Yorkshire, encodes a protein involved with component of the epithelial apical junction complex that is essential for the tight junction integrity. *INSR* identified in Landrace is a receptor tyrosine kinase that mediates an increase of glucose uptake activated by insulin.

**Table 1:** **Summary of overlaps between selective sweeps in European breeds and previous genomics studies on the signatures of selection**

**Association**	**Breed**	**Candidate genes**
**White coat color phenotype [** 4 **]**	Yorkshire	DNAJB5, RBBP4, PPRC1
	Landrace	ENSSSCG00000024845, ISOC1, KIAA1257, METTL13, TMTC1,
**Selective sweep in domestic pigs [** 36 **]**	Yorkshire	ABLIM1, BTBD11, C14orf174, C8orf38, CD68, CILP, CXADR, DNAJB5
	Landrace	INSR, METTL13, PCDHAC2, RIMS1, RPL35, SYNE1
**Domesticated pigs vs. wild boars [** 4 **]**	Yorkshire	BAI3, CCDC150, MPDU1, PGAP1, PKP4, ZNF638, ZNF804A
	Landrace	CNTFR, KBTBD12, LIMS3, PCDHAC2, PPFIA4, PRSS54, SF3B1
**Asian introgression [** 4 **]**	Yorkshire	AQP3, NOLC1, SYNE1, ZNF638
	Landrace	PPFIA4, STT3B

Moreover, we showed that 14 genes identified in this study overlap with the ‘domestication’ genes identified in previous studies [4,37] (Table 1). Four of them, including *BAI3*, *PKP4*, *PPFIA4*, and *PCDHAC2*, are associated with cell adhesion, and, five of them, including *LIMS3*, *BAI3*, *CNTFR*, *PKP4*, and *PCDHAC2*, are associated with signal transduction [38]. Of these genes, *CNTFR* provides an interesting evolutionary link between neuronal process and domestication. This gene encodes a member of the type 1 cytokine receptor family, which plays a critical role in neuronal cell survival, and may be associated with muscle strength and eating disorders [39]. Along with strongest sweep signals at *GRM7* on SSC13 and *GRM8* on SSC18, selection on those genes would provide the molecular evidence about the underlying mechanism involved in the alteration of the behavior phenotype during pig domestication.

It is to note that the highest signal of selection at 73.06 Mb on the SSC13 was identified in a previous study [4], suggesting *GHRL* (73.47-73.48 Mb) as a putative candidate under selection. We found no window around the locus was ranked within 1% of PBS bins. Instead we found that the locus at 73.65 Mb was ranked as 44,381^th^, top 0.006% of 7 M SNPs, by the *iHS* method. This observation can explain why *GHRL* was not identified in our study. The whole genome resequencing technology made it possible to detect a high level of novel genetic variation at high resolution where commercial probe-based SNP array platforms have a certain bias in probing SNPs with minor allele frequency around 0.5 [40]. Although those alleles with intermediate frequency are valuable resources for association studies and phylogenetic studies, they can have limited information of recent history of breed formation. As a result, the F_ST_ statistic averaged over all pairs of comparisons among 12 European breeds may be inappropriate to capture genetic variation that is fixed by directional positive selection. In fact, *GHRL* is located within the region showing strong signals of *iHS*. In this study, the high-resolution map of selective sweeps identified by using both PBS and *iHS* provides a comprehensive picture of genetic variation underlying pig domestication.

Additionally, out of 51 candidate loci involved in white coat color detected in a previous study [4], eight genes overlapped with this study. Five of these overlapping genes, including *DNAJB5*, *ISOC1*, *METTL13*, *PPRC1*, and *RBBP4*, are related with metabolic processes [38]. But, we found no overlap between genes in the contrast of belted and non-belted pigs [41].

### Selection on group III mGluR for tame behavior in domesticated breeds

By identifying genes harboring strong signals of directional selection, a new set of genes to be functionally validated beyond the list of QTLs were obtained. One of the most striking findings is a strong signal of artificial selection in *GRM7* and *GRM8*. These genes are included in the mGlu group III receptors that are linked to the inhibition of cyclic AMP cascade. In dogs, *GRM8* was detected to be positively selected using the method of identifying high divergence between indigenous dogs and wolves [42]. In mice, the knockdown of *GRM7* receptor mRNA levels reduced anxiety-associated behaviors, including stress levels and fear [43]. We suggest that selection on genetic variation in the mGlu III receptors might have played a critical role in the process of domestication that converts anxiety-associated aggressive behaviors of wild population to tame behaviors for the adaptation to the community. In fact, tail biting, a stress-induced behavior, is one of the most important issues in welfare of pigs. Tail biting has been observed in ~30% of European pigs, where the Yorkshire pigs are more likely to be victims of tail biting than Landrace pigs [44]. Further study is necessary to characterize the role of these genes in specific behavior of pigs.

### Artificial selection on the formation of pig breeds

It is well known that the European breeds have been domesticated from European wild boars followed by introgression in the 18-19th century of Asian haplotypes, which were derived from Asian domesticated breeds that have their origin in the Asian wild boar [7,17-19]. The main cause of introgression was the effort to introduce Asian-specific traits, i.e., production efficiency, into European breeds. Our analysis could also detect these Asian haplotypes, which resemble those seen in Asian wild boars, segregating in the European breeds by the *iHS* method. Out of 18 introgressed loci identified in a previous study [4], six genes overlapped in this study (Table 1). *ZNF638* is the most interesting candidate to note in that this gene encodes a nucleoplasimic protein associated with early regulator of adipogenesis that works as a transcription cofactor of CEBPs, controlling the expression of *PPARG*, and other proadipogenic genes [45]. This gene might shed light on what sort of genes were introgressed, and selected during domestication of European pigs. As Asian wild boars were used as an out-group population in our analysis, European-specific selection signals involving introgression could be pronounced. Therefore, introgression and admixture among breeds has contributed the structure of the genomes of domesticated breeds. Thus, caution is needed for interpreting significance of selection candidates, particularly for methods using haplotype structure.

## Conclusions

In this study the identification of putative sweeps based on high-depth whole genome NGS helps build an understanding of the effects of artificial selection during the process of animal domestication. Future studies are needed to fully characterize the process of complex admixture and introgression between pigs of different ancestry. To this end, a world-wide sampling of native breeds and wild boar genomes would be needed.

## Methods

### Ethics statement

For the pig experiment, the study protocol and standard operating procedures were reviewed and approved by the National Institute of Animal Science’s Institutional Animal Care and Use Committee (No. 2009–077, C-grade).

### Sample library preparation

Whole blood samples were collected from 7 males and 7 females of Landrace and 8 males and 8 females of Yorkshire (Large White) from the National institute of Animal Science, Korea and a set of muscle samples was collected from 3 males and 7 females of wild boars from the Southern part of Korea. Blood samples (10 ml) were drawn from the carotid artery and treated with heparin to prevent clotting. We randomly sheared 3 μg of genomic DNA using Covaris System to generate approximately 300-bp inserts. The fragmented DNA was end-repaired using T4 DNA polymerase and Klenow polymerase, and Illumina paired-end adaptor oligonucleotides were ligated to the ends. We analyzed the ligation mixture by electrophoresis on an agarose gel and purified fragments from specific gel slices. The purified DNA libraries were sequenced on a HiSeq2000 using recommended protocols from the manufacturer.

### Genotype calling and SNP calling

We processed paired-end sequence reads (~15X coverage of Illumina’s HiSeq 2000) which provided ~15X coverage of the reference pig genome (SusSc.10.2). Reads were aligned to the reference genome with the Burrows-Wheeler Aligner (BWA; version 0.6.1) using default parameters. Then, three open-source packages were used for downstream processing and variant calling; Picard Tools, SAMtools [46], and Genome Analysis ToolKit [47]. Specific options for SNP calling can be found in Additional file 18: Protocol.

Based on genotype likelihood values, we estimated the posterior probability of the minor allele frequency (*p*_*i,*_ i = 1,2,…, 2 k) in the sample of 2 k chromosomes, where *k* is the sample size of breeds [8]. The estimated values of *p*_*i*_, can then be used for population genetic inferences either by averaging over *p*_*i*_ or by using a Maximum Posteriori Probability (MAP) estimate of the sample allele frequency. SNP calling can proceed in a probabilistic fashion by choosing a cut-off for *p*_0_. And, the *p*_2k_ is so close to zero that it can be ignored because the definition of *p* as the minor allele frequency. We selected all sites with *p*_0_ < 0.05 to obtain SNPs with a probability > 95%. More details on the algorithm for estimating the posterior probability can be found in [48]. For each chromosome, we inferred haplotype phase information from all variable sites for the entire set of pig samples simultaneously using BEAGLE [49].

### Population structure

ADMIMXTURE was employed to analyse the population structure [29] . To mitigate the effects of LD, we pruned the markers according to the observed sample correlation using the ‘--indep-pairwise’ option of PLINK [30]. The result of ADMIXTURE was used to address relatedness within each breed by using RelateAdmix [31]. We further analyzed the population stratification based on the Multidimential scaling (MDS) analysis implemented in PLINK.

### Construction of a neutral genetic variation

For the estimation of population demography, we collected putative neutral sites with a uniform distribution (*p* = 0.001) from inter-genic regions, which are defined as variable sites more than 100 kb away from the start or end of any gene in the pig reference genome, and obtained folded site frequency spectra for wild boar, Yorkshire, and Landrace. Then we built a simple demographic model of three populations with two steps of population bottleneck leading to the two current breeds - the first bottleneck at the foundation of domesticated lineage and the second at the formation of Yorkshire and Landrace. We estimated the demographic parameters using *dadi* [50]. To avoid unrealistic estimations, we set the lower- and upper-boundaries of the prior distribution of the time of first domestication bottleneck, *T*_*b*_, to 5ky and 15ky, respectively. Using the first ten runs of converged parameters, we calculated standard deviations for the 11 parameters, and used them to set the upper- and lower-boundary of each parameter for the prior distribution of the subsequent runs. During the next 30 runs, we used the posterior of previous runs as a prior, but intentionally perturbed the starting parameters and checked to see if the parameter values had converged around the starting parameter values. We also compared this simple model and a model with another ancestral bottleneck prior to *T*_b_ (total 12 parameters). The log-likelihood for the model of two bottlenecks (−log(L) ≈ −11000) was much higher than that for the three-bottleneck model (−log(L) ≈ −15000). Under the estimated values of parameters (Additional file 19: Table S6), we obtained neutral chromosomes to construct the distribution of *PBS* of European breeds. Additionally, we computed the number of distinct haplotypes, *H* (in a window of 30 SNPs), from 50,000 replicates of neutral simulations without recombination by using Hudson’s *ms* [51]. Details of simulation commands can be found in the supplemental table (Additional file 19: Table S6).

### Calculation of population-specific branch score (PBS)

*F*_ST_ and other population differentiation indices are able to detect local selective sweeps but cannot indicate which lineage has experienced selection. The population branch statistic (PBS) has recently been proposed [8] to detect a significant change in allele or haplotype frequency along the lineage of one population after it diverged from other populations.

We estimated *F*_ST_ for a pair of populations by Hudson’s *K*_ST_ = 1 – (π_w_/π_t_) [52], where π_t_ is the nucleotide diversity (mean pairwise sequence difference) of total sequences and π_w_ is the mean nucleotide diversity of sequences sampled within the same population. The latter is given by (*n*_1_π_1_+ *n*_2_π_2_)/(*n*_1_ + *n*_2_) where *n*_*i*_ and π_*i*_ are the sample size and nucleotide diversity of population *i*, respectively. *F*_ST_ between population *i* and *j* is transformed into effective population divergence time *T*^*ij*^ [53].

$$ {T}^{ij} = - \log \left(1 - {F}_{\mathrm{ST}}\right) $$

For each bin, we calculated *T*^*ij*^ for three population pairs of Landrace (L), Yorkshire (Y), wild boar (W). The effective length of the branching leading to the Landrace population since the divergence from Yorkshire is then obtained as

$$ \mathrm{PB}{\mathrm{S}}_{\mathrm{L}} = \left({T}^{\mathrm{L}\mathrm{W}} + {T}^{\mathrm{L}\mathrm{Y}}\hbox{--} {T}^{\mathrm{WY}}\right)/2 $$

Similarly, the branch lengths for Yorkshire and wild boar, PBS_Y_ and PBS_W_ respectively, are obtained. Namely, a population-specific PBS value represents the amount of allele frequency change at a given locus in the history of a population since its divergence from the other two populations [8]. PBS was calculated for a sliding window of 200 SNPs with a step size of 50 SNPs, yielding 527,040 bins in total.

### Calculation of absolute integrated haplotype scores |*iHS*|

The statistical detection of sites under incomplete selective sweep was performed by calculating *iHS* statistics over individual SNP sites. The *iHS* is derived from the extended haplotype homozygosity (EHH) [54] that looks for unusually long haplotypes at the selected allele compared to non-selected allele background. To investigate signatures of possible directional selection after domestication, we operationally defined the derived allele in a domesticated lineage as the minor or non-existent allele in the wild boar at the same site. The derived allele defined in this way may not be the true derived (mutant) allele at many sites. However, as we will later rank the strengths of selection signal according to the absolute values of *iHS*, the mis-inference of ancestral/derived state may only slightly lower the detection power. This statistic is based on the integral of the observed decay of EHH (extended haplotype homozygosity) away from a focal allele until EHH reaches 0.05 [24]. This integrated EHH is computed for the ancestral (*iHH*_*A*_) at the core SNP (*iHH*_*D*_). The *iHS* statistic is given as the log ratio of *iHH*_*A*_ to *iHH*_*D*_ and its absolute value is standardized for each core-SNP frequency class to have mean of 0 and variance of 1. While *iHS* was calculated for all SNPs with MAF > 0.2 in each breed (7,202,005 sites in Yorkshire and 8,187,301 sites in Landrace), for the calculation of *EHH* all linked SNPs with any minor allele frequencies were used (i.e., the entire genomic set of 25,922,448 variable sites). The significance of the standardized *iHS* value was evaluated assuming that it follows normal distribution under the null model. All analysis was done by using *rehh* library [55] in R environment.

### Gene annotation

We took an outlier-approach to obtain the candidates of selection genes. First, each of the bins (*PBS*, Additional files 20 and 21: Table S7 and S8) or sites (|*iHS*|, Additional files 22 and 23: Table S9 and S10) that carries a strong selection signal is mapped to a gene (among 19,990 genes annotated in the pig genome). To have a high-resolution map, we limited the distance cutoff for gene annotations to be 1 kb: we define that a SNP belongs to a genes if it is located within the region defined by 1 kb upstream of transcription start site and 1 kb downstream of the transcription stop site. We choose the bin/site with the strongest signal if there was more than one bin/site assigned to one gene. Then, genes are ranked by the strength of the signal mapped to them. We obtained the top 1% of genes, producing 200 candidates for each breed and each method.

To assign associations with QTL, we used results of QTL mapping by previous studies that are compiled in the AnimalQTL database (www.animalgenome.org). The current release of the Pig QTLdb contains 8,402 QTLs from 356 publications. Each QTL is reported as an interval on the genetic map of the pig genome. We used mapped QTLs with sizes less than 5 cM (≈5Mbp) only. Redundant loci were excluded for further analysis. In total, 1,313 loci were obtained. After comparing the physical and genetic map of pig genome, the reported QTLs in the genome were obtained by interpolating their linkage map position via anchor markers (details in [34]), we assigned annotated genes to these QTLs, producing 4,055 QTL-candidate genes. These genes were further categorized into four classes, including “production”, “reproduction”, “exterior”, and “health”, according to the QTL database [34].

### Availability of supporting data

Samples that were sequenced were archived at the Sequence Read Archive (SRA) under the accession numbers: SAMN03031146-SAMN03031158, SAMN03031171-SAMN03031195 from SRP047260 and SRP052927.

## Additional files

Additional file 1: Figure S1. Admixture analysis for three lineages. A cross validation procedure (10-fold CV) shows that *K* = 2 and 3 exhibit a low cross-validation error compared to other K values (A). Each individual is represented by a vertical bar, which is partitioned into K colored segments that represent the individual’s estimated membership fractions with 10,000 *admixture* runs at K = 2 ~ 6 (B-F). For Admixture result for *K* = 2, Estimates of kinship coefficients (*k*), where k1 and k2 describe the fractions of the genome in which two individuals share 1 or 2 alleles IBD, from RelateAdmix, where dashed line depicts first cousins (*k* = 0.25), showing unrelated relationship between samples (G). Additional file 2: Figure S2. Distribution of population-specific branch length with relative nucleotide diversity. Given PBS values in Yorkshire and Landrace, log2-fold-ratio of relative nucleotide diversity in Yorkshire (π_Yorkshire_/π_wild boar_) and in Landrace (π_Landrace_/π_wild boar_) are depicted in (A) and (B), respectively. Additional file 3: Figure S3. Distribution of *iHS* scores between Yorkshire and Landrace. Low frequency derived allele (blue color) tend to have strong negative *iHS*_*unstd*_ values and high frequency derived allele (orange color) tend to have strong positive *iHS*_*unstd*_ values (A). The tendencies of allele frequency are neutralized after normalization (B). Additional file 4: Figure S4. Structure of sequence variation around focal sites of *PBS* signals in Yorkshire. Additional file 5: Figure S5. Structure of sequence variation around focal sites of *PBSS* signals in Landrace. Additional file 6: Figure S6. Structure of sequence variation around focal sites of *iHS* signals in Yorkshire. Additional file 7: Figure S7. Structure of sequence variation around focal sites of *iHS* signals in Landrace. Additional file 8: Figure S8. Structure of sequence variation around focal sites of *PBS* (A, C) and *iHS* (B, D) ranked at 1^th^ signals in Yorkshire (A, B) and Landrace (C, D). Only variable sites are shown in the alignment of sequences for wild boar (W), Yorkshire (Y) and Landrace (L). The ancestral and derived alleles are colored in orange and blue, respectively. Variable sites located up to 15 kb up-stream and 15 kb down-stream from the focal bin (for PBS being depicted with red region with vertical line)/site (for *iHS* being depicted with red arrow) are shown. The length of the entire block is variable as the number of variable sites included varies. At the region of the focal sites/bins, long haplotype homozygosity as well as strong between- and within-population differentiation of haplotypes showing patterns of directional selection and long haplotype. For *iHS* signals, haplotype on alternative allele background indicated in transparent blue. Additional file 9: Figure S9. Structure of sequence variation around focal sites of *PBS* (A, C) and *iHS* (B, D) ranked at 200^th^ signals in Yorkshire (A, B) and Landrace (C, D). Only variable sites are shown in the alignment of sequences for wild boar (W), Yorkshire (Y) and Landrace (L). The ancestral and derived alleles are colored in orange and blue, respectively. Variable sites located up to 15 kb up-stream and 15 kb down-stream from the focal bin (for PBS being depicted with red region with vertical line)/site (for *iHS* being depicted with red arrow) are shown. The length of the entire block is variable as the number of variable sites included varies. At the region of the focal sites/bins, long haplotype homozygosity as well as strong between- and within-population differentiation of haplotypes showing patterns of directional selection and long haplotype. For *iHS* signals, haplotype on alternative allele background indicated in transparent blue. Additional file 10: Figure S10. Genes detected based on population specific branch test and haplotype homozygosity test in Yorkshire and Landrace. To draw a simple presentation of potential candidates of selection (A), number of genes (5) shared between gene sets detected by PBS in Landrace and gene sets by *iHS* in Yorkshire was removed from the *ven diagram*. Significance of overlap was calculated by applying a bootstrap method (*n* = 100,000), in which two subset of genes were randomly sampled from all annotated genes (19,990) and the number of shared genes between them was counted. Additional file 11: Figure S11. Selection signals falling in QTL regions. The overlapping genes among all categories (A), and then they were merged into the breeds (B) and into the method (C) to show the proportion of sharing genes between breeds and methods, respectively. Significance for sharing between subsets of selection candidates and QTL-candidate genes was tested by the Hyper-geometric test. Additional file 12: Figure S12. Selection candidate genes associated with quantitative traits. Significance for sharing between subsets of selection candidates and QTL-candidates in the sub-QTL categories was tested by the Hyper-geometric test. Additional file 13: Table S1. Association of trait categories with candidate genes of artificial selection. Additional file 14: Table S2. List of genes with strong selection signals detected in both Landrace and Yorkshire. Additional file 15: Table S3. Yorkshire-specific strong selection-candidate genes associated with quantitative traits. Additional file 16: Table S4. Landrace-specific strong selection genes associated with quantitative traits. Additional file 17: Table S5. Strong selection-candidate genes associated with ‘Total Number of Born Alive (TNBA)’. Additional file 18:
**Protocol 1.** Genotype calling and SNP calling. Additional file 19: Table S6. Demographic parameter estimation. Additional file 20: Table S7. Top 1% of PBS in Yorkshire breed. Additional file 21: Table S8. Top 1% of PBS in Landrace breed. Additional file 22: Table S9. Top 1% of iHS in Yorkshire breed. Additional file 23: Table S10. Top 1% of iHS in Landrace breed.
