# Supplementary material for: A genome-wide scan for signatures of directional selection in domesticated pigs
Source: BMC Genomics. 2015 Feb 25;16(1):130. doi: 10.1186/s12864-015-1330-x (PMC4349229; doi:10.1186/s12864-015-1330-x)
Supplement: Additional file 1: Figure S1. — Admixture analysis for three lineages. A cross validation procedure (10-fold CV) shows that K = 2 and 3 exhibit a low cross-validation error compared to other K values (A). Each individual is represented by a vertical bar, which is partitioned into K colored segments that represent the individual’s estimated membership fractions with 10,000 admixture runs at K = 2 ~ 6 (B-F). For Admixture result for K = 2, Estimates of kinship coefficients (k), where k1 and k2 describe the fractions of the genome in which two individuals share 1 or 2 alleles IBD, from RelateAdmix, where dashed line depicts first cousins (k = 0.25), showing unrelated relationship between samples (G). [file 12864_2015_1330_MOESM1_ESM.docx]

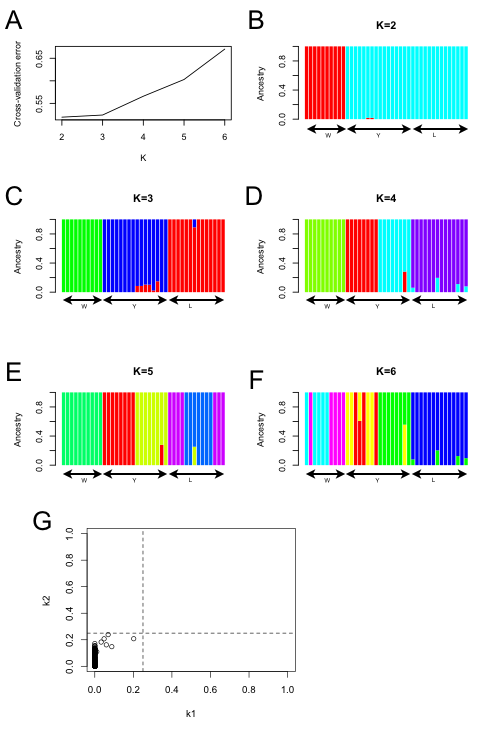


**Supplementary Figure S1**. Admixture analysis for three lineages. A cross validation procedure (10-fold CV) shows that *K* = 2 and 3 exhibit a low cross-validation error compared to other K values (A). Each individual is represented by a vertical bar, which is partitioned into K colored segments that represent the individual’s estimated membership fractions with 10,000 *admixture* runs at K=2~6 (B-F). For Admixture result for *K* = 2, Estimates of kinship coefficients (*k*), where k1 and k2 describe the fractions of the genome in which two individuals share 1 or 2 alleles IBD, from RelateAdmix, where dashed line depicts first cousins (*k*=0.25), showing unrelated relationship between samples (G).
