# Supplementary material for: A genome-wide scan for signatures of directional selection in domesticated pigs
Source: BMC Genomics. 2015 Feb 25;16(1):130. doi: 10.1186/s12864-015-1330-x (PMC4349229; doi:10.1186/s12864-015-1330-x)
Supplement: Additional file 2: Figure S2. — Distribution of population-specific branch length with relative nucleotide diversity. Given PBS values in Yorkshire and Landrace, log2-fold-ratio of relative nucleotide diversity in Yorkshire (πYorkshire/πwild boar) and in Landrace (πLandrace/πwild boar) are depicted in (A) and (B), respectively. [file 12864_2015_1330_MOESM2_ESM.docx]

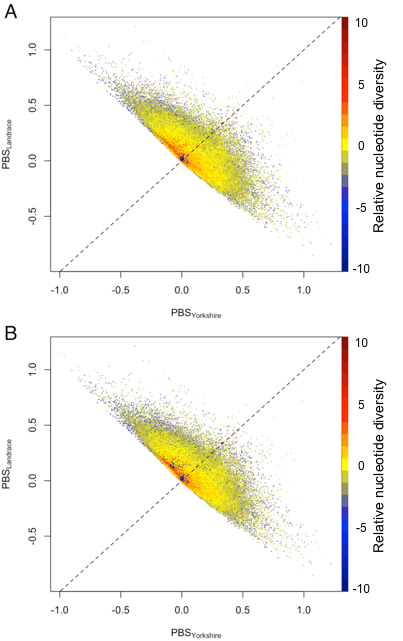


Supplementary Figure S2. Distribution of population-specific branch length with relative nucleotide diversity. Given PBS values in Yorkshire and Landrace, log2-fold-ratio of relative nucleotide diversity in Yorkshire (π_Yorkshire_ / π_wild boar_) and in Landrace (π_Landrace_ / π_wild boar_) are depicted in (A) and (B), respectively.
