# Supplementary material for: A genome-wide scan for signatures of directional selection in domesticated pigs
Source: BMC Genomics. 2015 Feb 25;16(1):130. doi: 10.1186/s12864-015-1330-x (PMC4349229; doi:10.1186/s12864-015-1330-x)
Supplement: Additional file 3: Figure S3. — Distribution of iHS scores between Yorkshire and Landrace. Low frequency derived allele (blue color) tend to have strong negative iHS unstd values and high frequency derived allele (orange color) tend to have strong positive iHS unstd values (A). The tendencies of allele frequency are neutralized after normalization (B). [file 12864_2015_1330_MOESM3_ESM.docx]

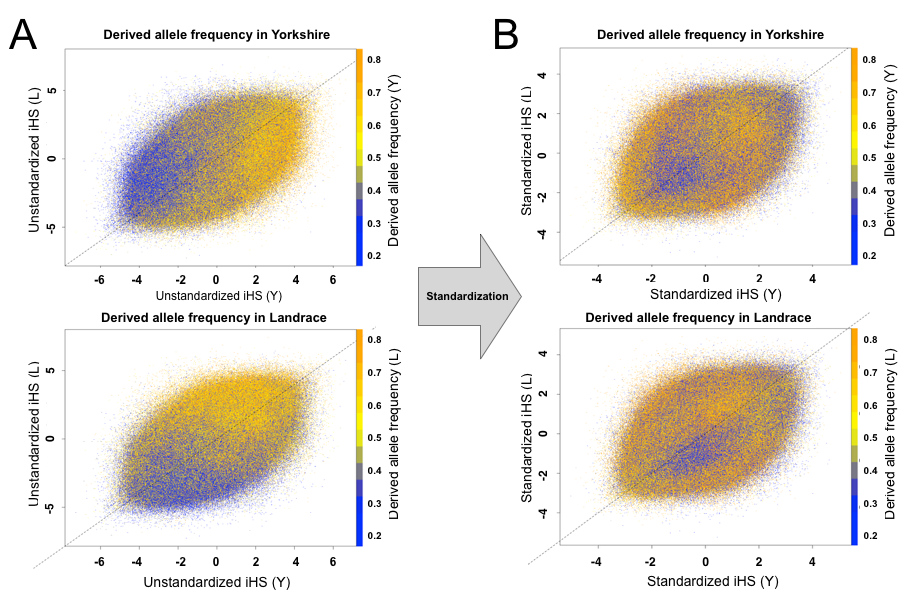


Supplementary Figure S3. Distribution of *iHS* scores between Yorkshire and Landrace. Low frequency derived allele (blue color) tend to have strong negative *iHS_unstd_* values and high frequency derived allele (orange color) tend to have strong positive *iHS_unstd_* values (A). The tendencies of allele frequency are neutralized after normalization (B).
