# Supplementary material for: A genome-wide scan for signatures of directional selection in domesticated pigs
Source: BMC Genomics. 2015 Feb 25;16(1):130. doi: 10.1186/s12864-015-1330-x (PMC4349229; doi:10.1186/s12864-015-1330-x)
Supplement: Additional file 4: Figure S4. — Structure of sequence variation around focal sites of PBS signals in Yorkshire. [file 12864_2015_1330_MOESM4_ESM.docx]

**Supplementary Figure S4**. Structure Genetic variations around the strongest selective sweep signals of PBS in Yorkshire. Heatmaps are arranged in the order of signal’s rank. Columns of each heatmap represent variables sites within Yorkshire (Y), Landrace (L), and Asian wild boar (W). To contrast between the Asian wild boar and the European pigs, high frequency allele in the Asian wild boar is colored in orange, and alternative allele is in blue color. Only variable sites up to 15kb up-stream and 15kb down-stream from the focal bin/site (red dashed box/red vertical line) are included in the heatmap. For *iHS* signals, haplotypes putatively carrying the positively selected allele (thus harboring low variation) reached the frequencies of 0.57 (B) and 0.69 (D) and the blocks of other haplotypes carrying the alternative allele are colored by transparent blue.

| PBS (Yorkshire) |
| --- |
| 1 |
| 2 |
| 3 |
| 4 |
| 5 |
| 6 |
| 7 |
| 8 |
| 9 |
| 10 |
| 11 |
| 12 |
| 13 |
| 14 |
| 15 |
| 16 |
| 17 |
| 18 |
| 19 |
| 20 |
| 21 |
| 22 |
| 23 |
| 24 |
| 25 |
| 26 |
| 27 |
| 28 |
| 29 |
| 30 |
| 31 |
| 32 |
| 33 |
| 34 |
| 35 |
| 36 |
| 37 |
| 38 |
| 39 |
| 40 |
| 41 |
| 42 |
| 43 |
| 44 |
| 45 |
| 46 |
| 47 |
| 48 |
| 49 |
| 50 |
| 51 |
| 52 |
| 53 |
| 54 |
| 55 |
| 56 |
| 57 |
| 58 |
| 59 |
| 60 |
| 61 |
| 62 |
| 63 |
| 64 |
| 65 |
| 66 |
| 67 |
| 68 |
| 69 |
| 70 |
| 71 |
| 72 |
| 73 |
| 74 |
| 75 |
| 76 |
| 77 |
| 78 |
| 79 |
| 80 |
| 81 |
| 82 |
| 83 |
| 84 |
| 85 |
| 86 |
| 87 |
| 88 |
| 89 |
| 90 |
| 91 |
| 92 |
| 93 |
| 94 |
| 95 |
| 96 |
| 97 |
| 98 |
| 99 |
| 100 |
| 101 |
| 102 |
| 103 |
| 104 |
| 105 |
| 106 |
| 107 |
| 108 |
| 109 |
| 110 |
| 111 |
| 112 |
| 113 |
| 114 |
| 115 |
| 116 |
| 117 |
| 118 |
| 119 |
| 120 |
| 121 |
| 122 |
| 123 |
| 124 |
| 125 |
| 126 |
| 127 |
| 128 |
| 129 |
| 130 |
| 131 |
| 132 |
| 133 |
| 134 |
| 135 |
| 136 |
| 137 |
| 138 |
| 139 |
| 140 |
| 141 |
| 142 |
| 143 |
| 144 |
| 145 |
| 146 |
| 147 |
| 148 |
| 149 |
| 150 |
| 151 |
| 152 |
| 153 |
| 154 |
| 155 |
| 156 |
| 157 |
| 158 |
| 159 |
| 160 |
| 161 |
| 162 |
| 163 |
| 164 |
| 165 |
| 166 |
| 167 |
| 168 |
| 169 |
| 170 |
| 171 |
| 172 |
| 173 |
| 174 |
| 175 |
| 176 |
| 177 |
| 178 |
| 179 |
| 180 |
| 181 |
| 182 |
| 183 |
| 184 |
| 185 |
| 186 |
| 187 |
| 188 |
| 189 |
| 190 |
| 191 |
| 192 |
| 193 |
| 194 |
| 195 |
| 196 |
| 197 |
| 198 |
| 199 |
| 200 |
