# Supplementary material for: A genome-wide scan for signatures of directional selection in domesticated pigs
Source: BMC Genomics. 2015 Feb 25;16(1):130. doi: 10.1186/s12864-015-1330-x (PMC4349229; doi:10.1186/s12864-015-1330-x)
Supplement: Additional file 9: Figure S9. — Structure of sequence variation around focal sites of PBS (A, C) and iHS (B, D) ranked at 200th signals in Yorkshire (A, B) and Landrace (C, D). Only variable sites are shown in the alignment of sequences for wild boar (W), Yorkshire (Y) and Landrace (L). The ancestral and derived alleles are colored in orange and blue, respectively. Variable sites located up to 15 kb up-stream and 15 kb down-stream from the focal bin (for PBS being depicted with red region with vertical line)/site (for iHS being depicted with red arrow) are shown. The length of the entire block is variable as the number of variable sites included varies. At the region of the focal sites/bins, long haplotype homozygosity as well as strong between- and within-population differentiation of haplotypes showing patterns of directional selection and long haplotype. For iHS signals, haplotype on alternative allele background indicated in transparent blue. [file 12864_2015_1330_MOESM9_ESM.docx]

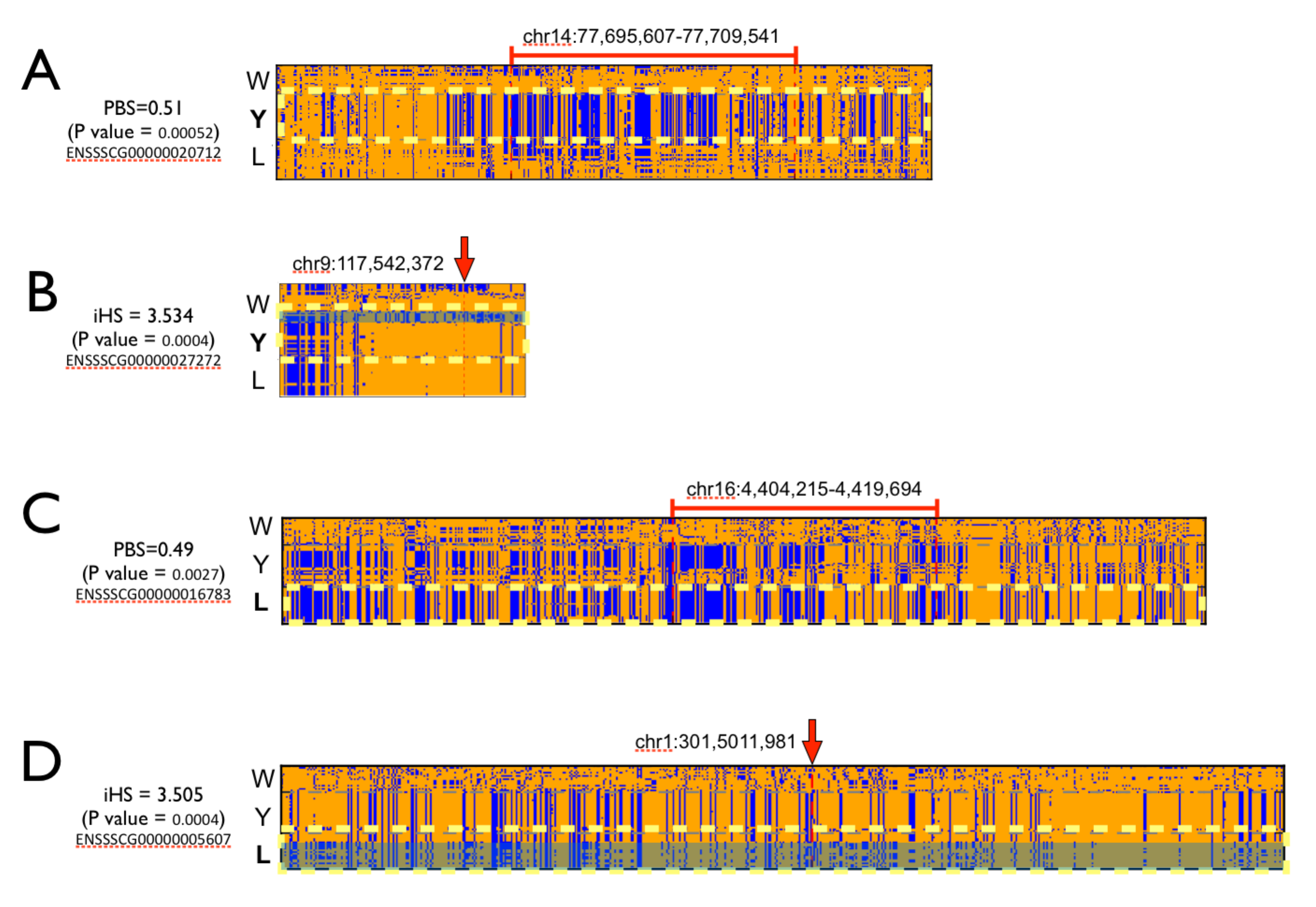


**Supplementary Figure S9**. Structure of sequence variation around focal sites of *PBS* (A, C) and *iHS* (B, D) ranked at 200^th^ signals in Yorkshire (A, B) and Landrace (C, D). Only variable sites are shown in the alignment of sequences for wild boar (W), Yorkshire (Y) and Landrace (L). The ancestral and derived alleles are colored in orange and blue, respectively. Variable sites located up to 15kb up-stream and 15kb down-stream from the focal bin (for PBS being depicted with red region with vertical line)/site (for *iHS* being depicted with red arrow) are shown. The length of the entire block is variable as the number of variable sites included varies. At the region of the focal sites/bins, long haplotype homozygosity as well as strong between- and within-population differentiation of haplotypes showing patterns of directional selection and long haplotype. For *iHS* signals, haplotype on alternative allele background indicated in transparent blue.
