# Supplementary material for: A genome-wide scan for signatures of directional selection in domesticated pigs
Source: BMC Genomics. 2015 Feb 25;16(1):130. doi: 10.1186/s12864-015-1330-x (PMC4349229; doi:10.1186/s12864-015-1330-x)
Supplement: Additional file 10: Figure S10. — Genes detected based on population specific branch test and haplotype homozygosity test in Yorkshire and Landrace. To draw a simple presentation of potential candidates of selection (A), number of genes (5) shared between gene sets detected by PBS in Landrace and gene sets by iHS in Yorkshire was removed from the ven diagram. Significance of overlap was calculated by applying a bootstrap method (n = 100,000), in which two subset of genes were randomly sampled from all annotated genes (19,990) and the number of shared genes between them was counted. [file 12864_2015_1330_MOESM10_ESM.docx]

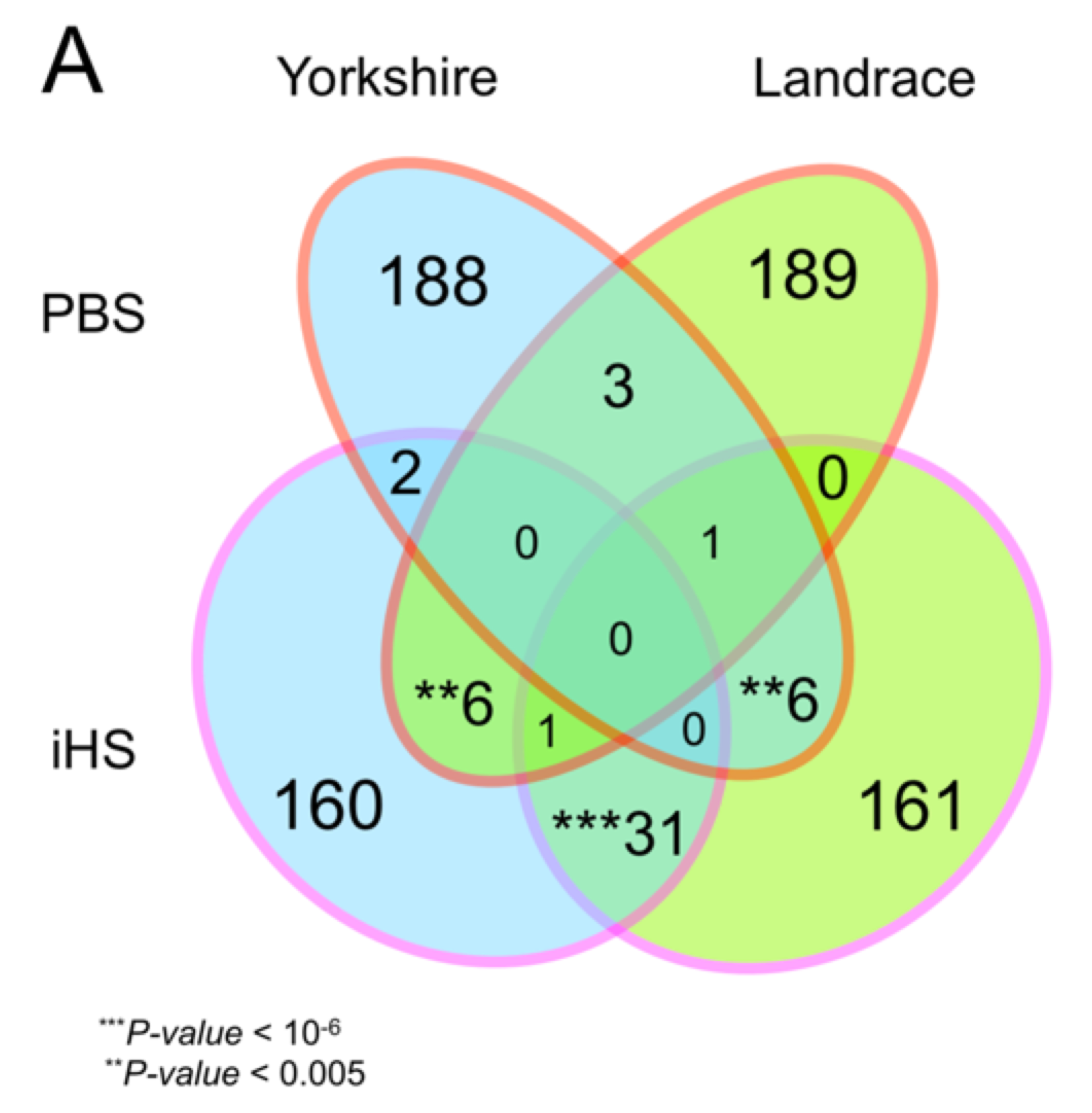


**Supplementary Figure S10**. Genes detected based on population specific branch test and haplotype homozygosity test in Yorkshire and Landrace. To draw a simple presentation of potential candidates of selection (A), number of genes (5) shared between gene sets detected by PBS in Landrace and gene sets by *iHS* in Yorkshire was removed from the *ven diagram*. Significance of overlap was calculated by applying bootstrap method (*n*=100,000), in which two subset of genes were randomly sampled from all annotated genes (19,969) and the number of shared genes between them was counted
