# Supplementary material for: A genome-wide scan for signatures of directional selection in domesticated pigs
Source: BMC Genomics. 2015 Feb 25;16(1):130. doi: 10.1186/s12864-015-1330-x (PMC4349229; doi:10.1186/s12864-015-1330-x)
Supplement: Additional file 11: Figure S11. — Selection signals falling in QTL regions. The overlapping genes among all categories (A), and then they were merged into the breeds (B) and into the method (C) to show the proportion of sharing genes between breeds and methods, respectively. Significance for sharing between subsets of selection candidates and QTL-candidate genes was tested by the Hyper-geometric test. [file 12864_2015_1330_MOESM11_ESM.docx]

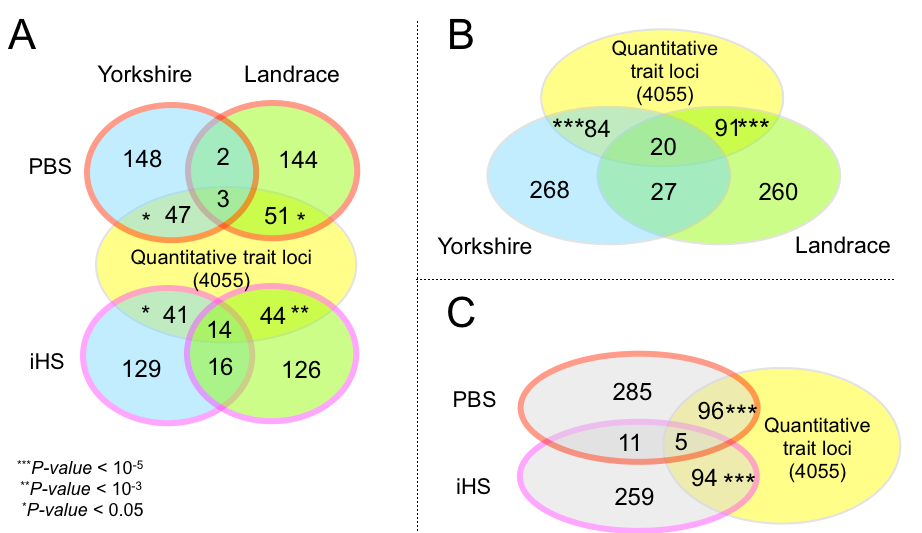


**Supplementary Figure S11.** Selection signals falling in QTL regions. The overlapping genes among all categories (A), and then they were merged into the breeds (B) and into the method (C) to show the proportion of sharing genes between breeds and methods, respectively. Significance for sharing between subset of selection candidates and QTL-candidate genes was tested by the Hyper-geometric test.
