# Supplementary material for: A genome-wide scan for signatures of directional selection in domesticated pigs
Source: BMC Genomics. 2015 Feb 25;16(1):130. doi: 10.1186/s12864-015-1330-x (PMC4349229; doi:10.1186/s12864-015-1330-x)
Supplement: Additional file 12: Figure S12. — Selection candidate genes associated with quantitative traits. Significance for sharing between subsets of selection candidates and QTL-candidates in the sub-QTL categories was tested by the Hyper-geometric test. [file 12864_2015_1330_MOESM12_ESM.docx]

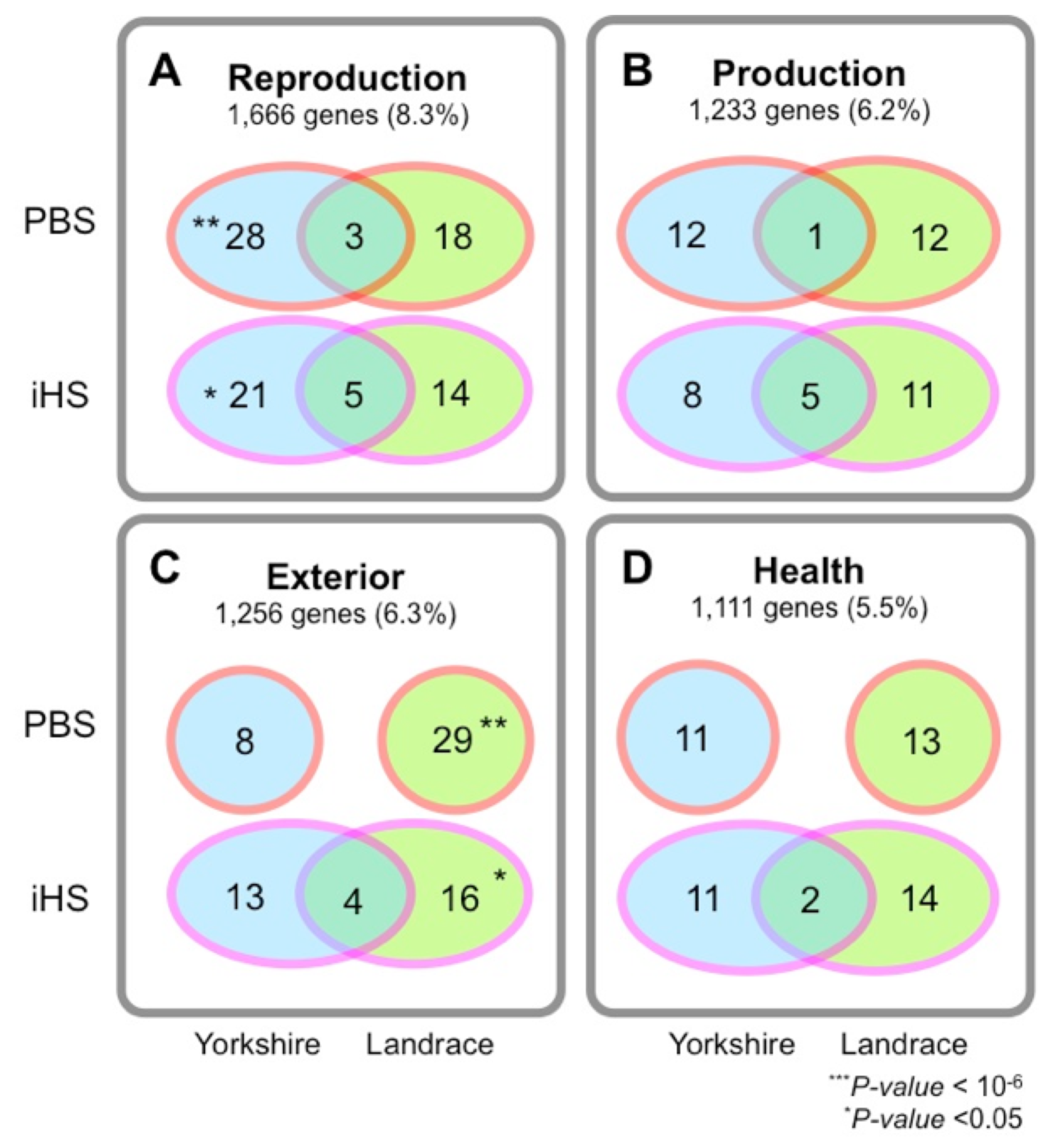


**Supplementary Figure S12**. Selection candidate genes associated with quantitative traits. Significance for sharing between subset of selection candidates and QTL-candidates in the sub-QTL categories was tested by the Hyper-geometric test.
