# Supplementary material for: A genome-wide scan for signatures of directional selection in domesticated pigs
Source: BMC Genomics. 2015 Feb 25;16(1):130. doi: 10.1186/s12864-015-1330-x (PMC4349229; doi:10.1186/s12864-015-1330-x)
Supplement: Additional file 13: Table S1. — Association of trait categories with candidate genes of artificial selection. [file 12864_2015_1330_MOESM13_ESM.docx]

**Supplementary Table S1**. **Association of trait categories with candidate genes of artificial selection**

| CATEGORY | NAME | YORKSHIRE | | LANDRACE | |
| --- | --- | --- | --- | --- | --- |
|  |  | **PBS** | **iHS** | **PBS** | **iHS** |
| Reproduction | Total number born alive (197) | ^a^15 | 3 | 1 | 1 |
|  | Mummified pigs (259) | 5 | ^a^10 | 5 | 4 |
|  | Age at puberty (65) | 0 | 0 | ^a^4 | ^a^2 |
| Exterior | Post-stress glucose level (1) | 0 | 0 | 0 | ^a^1 |
|  | Vertebra number (77) | 0 | ^a^11 | 1 | 0 |
|  | Gait score (overall) (81) | 0 | ^a^8 | 2 | 2 |
|  | Umbilical hernia (62) | 1 | ^a^5 | 0 | 4 |
|  | Hind leg conformation (228) | 0 | ^a^9 | 3 | 3 |
| Health | Spontaneous cell proliferation (1) | 0 | 0 | ^a^1 | 0 |
|  | Anti-O149 E.coli IgG level response (2) | ^a^1 | 0 | 0 | 0 |
|  | Sarcocystis miescheriana IgM levels (7) | ^a^2 | 0 | 0 | 0 |
|  | Red cell distribution width (36) | 0 | ^a^4 | 2 | 0 |
|  | HDL cholesterol (19) | 0 | ^a^3 | 1 | 0 |
|  | Body temperature (25) | ^a^4 | 0 | 0 | 0 |
|  | Post-stress mitogen induced IL-2 activity (76) | 0 | ^a^6 | 0 | 0 |
| Production | Days to 100 kg (7) | 0 | 0 | ^a^2 | ^a^3 |
|  | Body weight (26 weeks) (12) | 0 | 0 | ^a^2 | ^a^3 |
|  | Body weight (60 days) (2) | 0 | 0 | ^a^1 | 0 |
|  | Average feeding rate (2) | 0 | 0 | 0 | ^a^1 |
|  | Average daily gain (on test) (57) | 0 | ^a^4 | 2 | 3 |
|  | Body weight (25 weeks) (3) | ^a^1 | 0 | 0 | 0 |
|  | Average daily gain (birth-30 kg) (3) | 0 | 0 | ^a^1 | 0 |
|  | Lipid accretion rate (11) | 0 | 0 | 1 | ^a^2 |
|  | Tibia length (24) | ^a^3 | 1 | 0 | 0 |

*The number of genes associated with the corresponding sub-category is given in parenthesis. ^a^*P-* value (hyper-geometric test) remains significant after Bonferroni multiple testing correction (*p* < 0.0005).
