# Supplementary material for: A genome-wide scan for signatures of directional selection in domesticated pigs
Source: BMC Genomics. 2015 Feb 25;16(1):130. doi: 10.1186/s12864-015-1330-x (PMC4349229; doi:10.1186/s12864-015-1330-x)
Supplement: Additional file 14: Table S2. — List of genes with strong selection signals detected in both Landrace and Yorkshire. [file 12864_2015_1330_MOESM14_ESM.docx]

***Supplementary* Table S2. List of genes with strong selection signals detected in both Landrace and Yorkshire**

| **Category** | ***Test/**  **Breed** | **Rank** | **Chr.** | **Position** | **Gene** | **Description** | **Score** | ***P* value** |
| --- | --- | --- | --- | --- | --- | --- | --- | --- |
| Reproduction | *IHS/Y* | 8 | 10 | 71582617 | NET1 | Neuroepithelial Cell Transforming 1 | 4.480 | 7.45E-06 |
|  | *PBS/Y* | 8 | 13 | 79791125-79806036 | KBTBD12 | Kelch Repeat And BTB (POZ) Domain Containing 12 | 0.896 | 4.40E-06 |
|  | *IHS/Y* | 11 | 12 | 18039369 | LRRC37B | Leucine Rich Repeat Containing 37B | 4.462 | 8.10E-06 |
|  | *IHS/L* | 27 | 1 | 10376795 | TMEM181 | Transmembrane Protein 181 | 4.034 | 5.49E-05 |
|  | *IHS/Y* | 34 | 1 | 16318693 | SYNE1 | Spectrin Repeat Containing, Nuclear Envelope 1 | 4.055 | 5.01E-05 |
| Production | *IHS/Y* | 5 | 16 | 19045383 | PDZD2 | PDZ Domain Containing 2 | 4.606 | 4.11E-06 |
|  | *PBS/Y* | 8 | 13 | 79791125-79806036 | KBTBD12 | Kelch Repeat And BTB (POZ) Domain Containing 12 | 0.896 | 4.40E-06 |
|  | *IHS/Y* | 15 | 9 | 120879474 | CNTNAP2 | Contactin Associated Protein | 4.366 | 1.27E-05 |
|  | *IHS/L* | 16 | 7 | 32184338 | KHDRBS2 | KH Domain Containing, RNA Binding, Signal Transduction Associated 2 | 4.153 | 3.28E-05 |
|  | *IHS/L* | 44 | 7 | 31323455 | LRRC1 | Leucine Rich Repeat Containing 1 | 3.936 | 8.29E-05 |
| Exterior | *IHS/L* | 9 | 1 | 55588556 | BAI3 | Brain | 4.242 | 2.22E-05 |
|  | *IHS/Y* | 15 | 9 | 120879474 | CNTNAP2 | Contactin Associated Protein | 4.366 | 1.27E-05 |
|  | *PBS/L* | 15 | 2 | 148662195-148672881 | PCDHA6 | Protocadherin Alpha Subfamily C, 1 | 0.746 | 2.38E-04 |
|  | *IHS/Y* | 18 | 6 | 17163421 | RSPRY1 | Ring Finger And SPRY Domain Containing 1 | 4.279 | 1.88E-05 |
|  | *IHS/Y* | 71 | 5 | 7414628 | MICALL1 | Mical | -3.829 | 1.28E-04 |
| Health | *IHS/Y* | 5 | 16 | 19045383 | PDZD2 | PDZ Domain Containing 2 | 4.606 | 4.11E-06 |
|  | *PBS/L* | 17 | 1 | 16884735-16896580 | ESR1 | Estrogen Receptor 1; Nuclear Hormone Receptor | 0.745 | 2.40E-04 |
|  | *IHS/Y* | 34 | 1 | 16318693 | SYNE1 | Spectrin Repeat Containing, Nuclear Envelope 1 | 4.055 | 5.01E-05 |
|  | *IHS/Y* | 112 | 9 | 148285411 | PLXNA2 | Plexin A2 | 3.725 | 1.95E-04 |
| Non-QTL | *PBS/Y* | 1 | 14 | 78799172-78822942 | COL13A1 | Collagen, Type XIII, Alpha 1 | 1.117 | <1E-06 |
|  | *IHS/Y* | 2 | 1 | 56512582 | COL19A1 | Collagen, Type XIX, Alpha 1 | 4.665 | 3.08E-06 |
|  | *IHS/Y* | 4 | 14 | 112022200 | PCGF5 | Polycomb Group Ring Finger 5 | 4.623 | 3.79E-06 |
|  | *PBS/Y* | 4 | 14 | 135891593-135902759 | ABLIM1 | Actin Binding LIM Protein 1 | 0.990 | 2.00E-06 |
|  | *PBS/Y* | 12 | 15 | 121405326-121414701 | ADAM23 | ADAM Metallopeptidase Domain 23 | 0.828 | 1.06E-05 |
|  | *IHS/L* | 18 | 5 | 45157831 | BICD1 | Bicaudal D Homolog 1 (Drosophila) | 4.138 | 3.51E-05 |
|  | *IHS/L* | 19 | 18 | 22838276 | GRM8 | Glutamate Receptor, Metabotropic 8 | 4.126 | 3.68E-05 |
|  | *IHS/Y* | 20 | 10 | 14017974 | WDR64 | WD Repeat Domain 64 | 4.236 | 2.28E-05 |
|  | *PBS/Y* | 20 | 14 | 77287300-77320685 | MYPN | Myopalladin | 0.791 | 2.20E-05 |
|  | *IHS/Y* | 21 | 12 | 55568509 | CNTROB | Centrobin, Centrosomal BRCA2 Interacting Protein | 4.212 | 2.53E-05 |

*Highest rank signal for each gene in Landrace or in Yorkshire and by *PBS* or by *iHS* is listed.
