# Supplementary material for: A genome-wide scan for signatures of directional selection in domesticated pigs
Source: BMC Genomics. 2015 Feb 25;16(1):130. doi: 10.1186/s12864-015-1330-x (PMC4349229; doi:10.1186/s12864-015-1330-x)
Supplement: Additional file 15: Table S3. — Yorkshire-specific strong selection-candidate genes associated with quantitative traits. [file 12864_2015_1330_MOESM15_ESM.docx]

***Supplementary* Table S3**. **Yorkshire-specific strong selection-candidate genes associated with quantitative traits**

| **Category** | **Test** | **Rank** | **Chr.** | **Position** | **Gene** | **Description** | **Score** | **P value** |
| --- | --- | --- | --- | --- | --- | --- | --- | --- |
| Reproduction | *PBS* | 3 | 13 | 80710266-80748093 | TXNRD3 | Thioredoxin reductase 3 | 1.038 | 2.00E-06 |
|  | *PBS* | 7 | 13 | 79887098-79916292 | RUVBL1 | Ruvb like 1 (E. coli) | 0.922 | 4.40E-06 |
|  | *PBS* | 16 | 13 | 80980118-81003195 | ACPP | Acid phosphatase, prostate | 0.816 | 1.38E-05 |
|  | *PBS* | 19 | 18 | 53735531-53746245 | TNS3 | Tensin 3 | 0.793 | 2.14E-05 |
|  | *PBS* | 27 | 1 | 303051062-303063821 | WDR34 | WD repeat domain 34 | 0.738 | 4.56E-05 |
| Production | *IHS* | 6 | 16 | 19045383 | PDZD2 | PDZ domain containing 2 | 4.606 | 4.11E-06 |
|  | *PBS* | 7 | 13 | 79887098-79916292 | RUVBL1 | Ruvb like 1 (E. coli) | 0.922 | 4.40E-06 |
|  | *PBS* | 27 | 1 | 303051062-303063821 | WDR34 | WD repeat domain 34 | 0.738 | 4.56E-05 |
|  | *PBS* | 39 | 13 | 80062344-80083380 | MGLL | Monoglyceride lipase | 0.699 | 7.34E-05 |
|  | *IHS* | 53 | 9 | 19291728 | ANKRD42 | Ankyrin repeat domain 42 | -3.929 | 8.52E-05 |
| Exterior | *IHS* | 9 | 13 | 214833775 | BACE2 | Beta-site APP-cleaving enzyme 2 | 4.477 | 7.58E-06 |
|  | *PBS* | 15 | 6 | 65908053-65915406 | PLOD1 | Procollagen-lysine 1,2-oxoglutarate-dioxygenase 1 | 0.817 | 1.32E-05 |
|  | *IHS* | 26 | 5 | 2155035 | PARVB | Parvin, beta | 4.100 | 4.13E-05 |
|  | *PBS* | 35 | 5 | 6588807-6603886 | CBY1 | Chibby homolog 1 (Drosophila) | 0.712 | 5.96E-05 |
|  | *PBS* | 49 | 5 | 4379207-4387141 | POLR3H | Polymerase (RNA) III (DNA directed) polypeptide H (22.9kd) | 0.683 | 8.62E-05 |
| Health | *IHS* | 6 | 16 | 19045383 | PDZD2 | PDZ domain containing 2 | 4.606 | 4.11E-06 |
|  | *IHS* | 9 | 13 | 214833775 | BACE2 | Beta-site APP-cleaving enzyme 2 | 4.477 | 7.58E-06 |
|  | *IHS* | 26 | 5 | 2155035 | PARVB | Parvin | 4.100 | 4.13E-05 |
|  | *PBS* | 35 | 5 | 6588807-6603886 | CBY1 | Chibby homolog 1 (Drosophila); | 0.712 | 5.96E-05 |
|  | *PBS* | 36 | 6 | 33286238-33300861 | SHCBP1 | SHC SH2-domain binding protein 1 | 0.708 | 6.30E-05 |
| Non-QTL | *IHS* | 1 | 13 | 70854033 | GRM7 | Glutamate receptor, metabotropic 7 | 4.838 | 1.31E-06 |
|  | *PBS* | 2 | 6 | 88524842-88539756 | HEYL | Hairy/split related with YRPW motif like | 1.054 | 2.00E-06 |
|  | *IHS* | 3 | 1 | 280824616 | EPB41L4B | Erythrocyte membrane protein band 4.1 like 4B | -4.625 | 3.75E-06 |
|  | *PBS* | 5 | 8 | 45769367-45790311 | KLHL2 | Kelch-like 2, Mayven (Drosophila) | 0.961 | 2.60E-06 |
|  | *PBS* | 6 | 8 | 45913746-45937016 | TMEM192 | Transmembrane protein 192 | 0.951 | 2.60E-06 |
|  | *IHS* | 7 | 16 | 27731591 | OXCT2 | 3-oxoacid CoA transferase 2 | 4.570 | 4.87E-06 |
|  | *PBS* | 9 | 14 | 37623390-37643095 | TESC | Tescalcin | 0.890 | 5.80E-06 |
|  | *IHS* | 10 | 5 | 22349026 | OR6C2 | **Olfactory receptor, family 6, subfamily C, member 2 (OR6C2), mrna | -4.465 | 7.99E-06 |
|  | *PBS* | 10 | 1 | 139587332-139615362 | Protein  metabolism | (Ipr009001/ipr004160/ipr009000) | 0.856 | 5.80E-06 |
|  | *PBS* | 11 | 6 | 88471816-88495392 | PABPC4 | Poly(A) binding protein, cytoplasmic 4 (inducible form) | 0.836 | 9.20E-06 |

* Highest rank signal either in *PBS* or in *iHS* is shown.

**Annotated as pseudogene in pig.
