# Supplementary material for: A genome-wide scan for signatures of directional selection in domesticated pigs
Source: BMC Genomics. 2015 Feb 25;16(1):130. doi: 10.1186/s12864-015-1330-x (PMC4349229; doi:10.1186/s12864-015-1330-x)
Supplement: Additional file 16: Table S4. — Landrace-specific strong selection genes associated with quantitative traits. [file 12864_2015_1330_MOESM16_ESM.docx]

***Supplementary* Table S4**. **Landrace-specific** **strong selection** **genes associated with quantitative traits**

| **Category** | ***Test** | **Rank** | **Chr.** | **Position** | **Gene** | **Description** | **Score** | ***P* value** |
| --- | --- | --- | --- | --- | --- | --- | --- | --- |
| Reproduction | *IHS* | 12 | 6 | 17689365 | GPR97 | G protein-coupled receptor 97; Orphan receptor | 4.186 | 2.84E-05 |
|  | *IHS* | 24 | 7 | 48104062 | SLC25A27 | Solute carrier family 25, member 27 | 4.064 | 4.82E-05 |
|  | *IHS* | 28 | 7 | 47596497 | CYP39A1 | Cytochrome P450, family 39, subfamily A, polypeptide 1 | -4.033 | 5.51E-05 |
|  | *PBS* | 50 | 15 | 83456476-83477902 | STK39 | Serine threonine kinase 39 (STE20/SPS1 homolog, yeast) | 0.655 | 5.42E-04 |
|  | *IHS* | 56 | 13 | 210839405 | TTC3 | Tetratricopeptide repeat domain 3-like | 3.863 | 1.12E-04 |
| Production | *PBS* | 12 | 1 | 298152654-298190781 | DENND1A | DENN/MADD domain containing 1A | 0.775 | 1.77E-04 |
|  | *IHS* | 24 | 7 | 48104062 | SLC25A27 | Solute carrier family 25, member 27 | 4.064 | 4.82E-05 |
|  | *IHS* | 46 | 8 | 11389290 | LDB2 | LIM domain binding 2 | 3.929 | 8.54E-05 |
|  | *PBS* | 64 | 2 | 132185741-132196740 | CEP120 | Centrosomal protein 120kDa | 0.629 | 6.89E-04 |
|  | *PBS* | 66 | 1 | 299954777-299966638 | MAPKAP1 | Mitogen-activated protein kinase associated protein 1 | 0.628 | 7.02E-04 |
| Exterior | *PBS* | 12 | 1 | 298152654-298190781 | DENND1A | DENN/MADD domain containing 1A | 0.775 | 1.77E-04 |
|  | *PBS* | 41 | 6 | 84032634-84049671 | CSMD2 | CUB and Sushi multiple domains 2 | 0.678 | 4.44E-04 |
|  | *PBS* | 43 | 6 | 82740840-82752218 |  |  | 0.670 | 4.79E-04 |
|  | *IHS* | 45 | 12 | 6506554 | CD300LD | CD300 molecule-like family member d | 3.932 | 8.44E-05 |
|  | *PBS* | 47 | 6 | 82971781-82997979 | ZBTB8A | Zinc finger and BTB domain containing 8A | 0.659 | 5.21E-04 |
| Health | *IHS* | 5 | 7 | 119802194 | CATSPERB | Cation channel, sperm-associated, beta | 4.557 | 5.18E-06 |
|  | *IHS* | 24 | 7 | 48104062 | SLC25A27 | Solute carrier family 25, member 27 | 4.064 | 4.82E-05 |
|  | *PBS* | 28 | 1 | 16493807-16509215 | SYNE1 | Spectrin repeat containing, nuclear envelope 1 | 0.701 | 3.50E-04 |
|  | *IHS* | 35 | 18 | 26001343 | CADPS2 | Ca++-dependent secretion activator 2 | -3.998 | 6.38E-05 |
|  | *IHS* | 40 | 12 | 15599760 | TANC2 | tetratricopeptide repeat, ankyrin repeat and coiled-coil containing 2 | 3.949 | 7.86E-05 |
| Non-QTL | *IHS* | 1 | 14 | 7884445 | TNFRSF10C | Tumor necrosis factor receptor superfamily, member 10c, decoy without an intracellular domain | 4.717 | 2.39E-06 |
|  | *PBS* | 1 | 14 | 122811662-122831617 | C10orf76 | UPF0668 protein C10orf76 | 1.149 | 6.20E-06 |
|  | *IHS* | 2 | 8 | 126943619 | BDH2 | 3-hydroxybutyrate dehydrogenase, type 2 | 4.633 | 3.61E-06 |
|  | *IHS* | 3 | 8 | 126943619 |  | Putative protein NHEDC1-like 1 | 4.633 | 3.61E-06 |
|  | *PBS* | 3 | 9 | 125626521-125640508 | VAMP4 | Vesicle-associated membrane protein 4; | 0.997 | 2.68E-05 |
|  | *IHS* | 4 | 6 | 137841830 | ATG4C | ATG4 autophagy related 4 homolog C (S. cerevisiae) | 4.596 | 4.30E-06 |
|  | *PBS* | 5 | 9 | 125667471-125678461 | METTL13 | methyltransferase like 13 | 0.853 | 8.76E-05 |
|  | *IHS* | 6 | 14 | 64930080 | GALNT2 | UDP-N-acetyl-alpha-D-galactosamine:polypeptide N-acetylgalactosaminyltransferase 2 (GalNAc-T2) | 4.537 | 5.71E-06 |
|  | *PBS* | 6 | 5 | 47342745-47358410 | TMTC1 | Transmembrane and tetratricopeptide repeat containing 1 | 0.841 | 9.84E-05 |
|  | *IHS* | 7 | 9 | 2459081 | PPFIBP2 | PTPRF interacting protein, binding protein 2 (liprin beta 2) | 4.448 | 8.65E-06 |

* Highest rank signal either in *PBS* or in *iHS* is shown.
