# Supplementary material for: A genome-wide scan for signatures of directional selection in domesticated pigs
Source: BMC Genomics. 2015 Feb 25;16(1):130. doi: 10.1186/s12864-015-1330-x (PMC4349229; doi:10.1186/s12864-015-1330-x)
Supplement: Additional file 17: Table S5. — Strong selection-candidate genes associated with ‘Total Number of Born Alive (TNBA)’. [file 12864_2015_1330_MOESM17_ESM.docx]

**Supplementary Table S5. Strong selection-candidate genes associated with ‘Total Number of Born Alive (TNBA)’**

| Rank | Test/  Breed | Chr. | Position | Gene | Description | Score | *p*-value |
| --- | --- | --- | --- | --- | --- | --- | --- |
| 3 | *PBS/Y* | 13 | 80710266-80748093 | TXNRD3 | Thioredoxin reductase 3 | 1.038 | 2.00E-06 |
| 7 | *PBS/Y* | 13 | 79887098-79916292 | RUVBL1 | RuvB-like 1 (E. coli) | 0.922 | 4.40E-06 |
| 8 | *PBS/Y* | 13 | 79791125-79806036 | KBTBD12 | Kelch repeat and BTB (POZ) domain containing 12 | 0.896 | 4.40E-06 |
| 16 | *PBS/Y* | 13 | 80980118-81003195 | ACPP | Acid phosphatase, prostate | 0.816 | 1.38E-05 |
| 30 | *PBS/Y* | 13 | 79401518-79446100 | C3orf37 | UPF0361 protein DC12 | 0.723 | 5.36E-05 |
| 31 | *PBS/Y* | 13 | 79401518-79446100 | H1FX | H1 histone family, member X | 0.723 | 5.36E-05 |
| 32 | *PBS/Y* | 13 | 79401518-79446100 | COPG | Coatomer protein complex, subunit gamma | 0.723 | 5.36E-05 |
| 37 | *PBS/Y* | 13 | 79593489-79633270 | GATA2 | GATA binding protein 2 | 0.705 | 6.76E-05 |
| 38 | *IHS/Y* | 6 | 69919216 | CROCC | Ciliary rootlet coiled-coil, rootletin | 4.036 | 4.27E+00 |
| 38 | *PBS/Y* | 13 | 79593489-79633270 | EEFSEC | Eukaryotic elongation factor, selenocysteine-tRNA-specific | 0.705 | 6.76E-05 |
| 39 | *PBS/Y* | 13 | 80062344-80083380 | MGLL | Monoglyceride lipase | 0.699 | 7.34E-05 |
| 65 | *PBS/Y* | 13 | 80166637-80196645 | MCM2 | Minichromosome maintenance complex component 2 | 0.663 | 1.05E-04 |
| 66 | *PBS/Y* | 3 | 116901226-116910947 | ALK | Anaplastic lymphoma receptor tyrosine kinas | 0.661 | 1.05E-04 |
| 67 | *PBS/Y* | 13 | 79414386-79454822 | RAB7A | RAB7A, member RAS oncogene family | 0.661 | 1.05E-04 |
| 86 | *PBS/Y* | 13 | 82897524-82919565 | RYK | RYK receptor-like tyrosine kinase | 0.634 | 1.50E-04 |
| 87 | *PBS/L* | 13 | 79756943-79786077 | KBTBD12 | Kelch repeat and BTB (POZ) domain containing 12 | 0.585 | 1.08E-03 |
| 133 | *IHS/L* | 2 | 119938305 | TMEM232 | Transmembrane protein 232 | 3.619 | 3.53E+00 |
| 143 | *PBS/Y* | 13 | 80608051-80699367 | PLXNA1 | Plexin A1 | 0.564 | 3.29E-04 |
| 162 | *PBS/L* | 13 | 79746631-79779646 | SEC61A1 | Sec61 alpha 1 subunit (S. cerevisiae) | 0.509 | 2.26E-03 |
| 187 | *PBS/L* | 3 | 112996208-113058084 | RASGRP3 | RAS guanyl releasing protein 3 (calcium and DAG-regulated) | 0.496 | 2.58E-03 |
| 194 | *PBS/L* | 1 | 16637716-16651582 | ESR1 | Estrogen receptor 1 | 0.492 | 2.67E-03 |
