# Supplementary material for: A genome-wide scan for signatures of directional selection in domesticated pigs
Source: BMC Genomics. 2015 Feb 25;16(1):130. doi: 10.1186/s12864-015-1330-x (PMC4349229; doi:10.1186/s12864-015-1330-x)
Supplement: Additional file 18: — Protocol 1. Genotype calling and SNP calling. [file 12864_2015_1330_MOESM18_ESM.docx]

**Supplementary Protocol**

**Genotype calling and SNP calling**

Paired-end sequence reads (~15X coverage) were mapped to the reference pig genome (SusSc.10.2) with the Burrows-Wheeler Aligner (BWA; version 0.6.1) using default setting. Three open-source packages were used for downstream processing and variant calling; Picard Tools, SAMtools [^1^](#_ENREF_1), and Genome analysis toolkit [^2^](#_ENREF_2). When a sample consisted of several BAM files of multiple lanes, we processed each BAM independently and then merged them into one BAM file after step 9): calculating covariates after recalibration and realignment. For SNP calling, BAM file of step 9) was used as an input of ANGSD [^3^](#_ENREF_3).

**1) Align both pairs of FASTQ files**

bowtie2 -p 8

-x /disk/Pigref/Pigref

-1 /disk/S1/S1.1.LANE1.fastq.gz

-2 /disk/S1/S1.2.LANE1.fastq.gz

-S /disk/S1/S1_LANE1.sam

**2) Reference genome soring (SAM to BAM)**

picard-tools-1.66/AddOrReplaceReadGroups.jar

INPUT=/disk/S1/S1_LANE1.sam

OUTPUT=/disk/S1/S1_LANE1_RG.bam

SORT_ORDER=coordinate

RGID=S1_LANE1 RGLB=S1_LANE1 RGPL=illumina RGPU=non

RGSM=S1_LANE1 VALIDATION_STRINGENCY=LENIENT

**3) Mark duplicates after alignment**

picard-tools-1.66/MarkDuplicates.jar

INPUT=/disk/S1/S1_LANE1_RG.bam

OUTPUT=/disk/S1/S1_LANE1_RG_DU.bam

METRICS_FILE=/disk/S1/S1_LANE1_RG_DU.metrics

REMOVE_DUPLICATES=true

ASSUME_SORTED=true VALIDATION_STRINGENCY=LENIENT

**4) Fix mates after realignment**

picard-tools-1.66/FixMateInformation.jar

INPUT=/disk/S1/S1_LANE1_RG_DU.bam

OUTPUT=/disk/S1/S1_LANE1_RG_DU_FIX.bam

SORT_ORDER=coordinate VALIDATION_STRINGENCY=LENIENT

samtools index /disk/S1/S1_LANE1_RG_DU_FIX.bam

picard-tools-1.66/FixMateInformation.jar

**5) Emits intervals for Local Indel Realigner**

GenomeAnalysisTK-1.5-31/GenomeAnalysisTK.jar

-T RealignerTargetCreator

-R /disk/Pigref/Pigref.fasta

-I /disk/S1/S1_LANE1_RG_DU_FIX.bam

-o /disk/S1/S1_LANE1_RG_DU_FIX_Realign.intervals

**6) Performs local realignment of reads to correct misalignments due to the presence of indels**

GenomeAnalysisTK-1.5-31/GenomeAnalysisTK.jar

-T IndelRealigner

-R /disk/Pigref/Pigref.fasta

-I /disk/S1/S1_LANE1_RG_DU_FIX.bam

-o /disk/S1/S1_LANE1_RG_DU_FIX_Realign.bam

-targetIntervals /disk/S1/S1_LANE1_RG_DU_FIX_Realign.intervals

**7) Calculate covariates before realignment**

GenomeAnalysisTK-1.5-31/GenomeAnalysisTK.jar

-T CountCovariates

-R /disk/Pigref/Pigref.fasta

-I /disk/S1/S1_LANE1_RG_DU_FIX_Realign.bam

–recalFile /disk/S1/S1_LANE1_RG_DU_FIX_Realign.recal_data.csv

-cov ReadGroupCovariate -cov QualityScoreCovariate

-cov CycleCovariate -cov DinucCovariate

-knownSites /disk/Pigref/korean_native_pig_only_snp.vcf

--default_platform illumina -U ALLOW_SEQ_DICT_INCOMPATIBILITY

**8) Recalibrate mate fixed and realigned alignment**

GenomeAnalysisTK-1.5-31/GenomeAnalysisTK.jar

-T TableRecalibration

-R /disk/Pigref/Pigref.fasta

-I /disk/S1/S1_LANE1_RG_DU_FIX_Realign.bam

-recalFile /disk/S1/S1_LANE1_RG_DU_FIX_Realign.recal_data.csv

-o /disk/S1/S1_LANE1_RECAL.bam

--default_platform illumina

**9) Calculate covariates after realignment and recalibration**

GenomeAnalysisTK-1.5-31/GenomeAnalysisTK.jar

-T CountCovariates

-R /disk/Pigref/Pigref.fasta

-I /disk/S1/S1_LANE12_RG_DU_FIX_Realign.bam

-recalFile /disk/S1/S1_LANE12_RG_DU_FIX_Realign.recal_data.csv

-cov ReadGroupCovariate -cov QualityScoreCovariate

-cov CycleCovariate -cov DinucCovariate

-knownSites /disk/Pigref/korean_native_pig_only_snp.vcf

--default_platform illumina -U ALLOW_SEQ_DICT_INCOMPATIBILITY

**10) Merge multiple lanes to one**

picard-tools-1.66/MergeSamFiles.jar

I=/disk/S1/S1_LANE1_RECAL.bam

…

I=/disk/S1/S1_LANE1*n*_RECAL.bam

OUTPUT=/disk/S1/S1_LANE1Merge.bam SORT_ORDER=coordinate

ASSUME_SORTED=ture USE_THREADING=true VALIDATION_STRINGENCY=SILENT

**11) Replaces all read groups in the input file with a new read group**

picard-tools-1.66/AddOrReplaceReadGroups.jar

INPUT=/disk/S1/S1_LANE1Merge.bam

OUTPUT=/disk/S1/S1_LANE1Merge_RG.bam

SORT_ORDER=coordinate RGID=S1 RGLB=S1 RGPL=illumina RGPU=non

RGSM=S1 VALIDATION_STRINGENCY=SILENT

samtools index /disk/S1/S1_LANE1Merge_RG.bam

**12) A variant call which unifies the approaches of several disparate callers**

GenomeAnalysisTK-1.5-31/GenomeAnalysisTK.jar

-T UnifiedGenotyper

-R /disk/Pigref/Pigref.fasta

-I /disk/S1/S1_LANE1Merge_RG.bam

-o /disk/S1/S1.raw.vcf

--dbsnp /disk/Pigref/korean_native_pig_only_snp.vcf

-glm BOTH

**13) Select variants from a VCF source**

GenomeAnalysisTK-1.5-31/GenomeAnalysisTK.jar

-R /disk/Pigref/Pigref.fasta

-T SelectVariants

--variant /disk/S1/S1.raw.vcf

-o /disk/S1/snp_S1.raw.vcf -selectType SNP

GenomeAnalysisTK-1.5-31/GenomeAnalysisTK.jar

-R /disk/Pigref/Pigref.fasta

-T SelectVariants

--variant /disk/S1/S1.raw.vcf

-o /disk/S1/indel_S1.raw.vcf -selectType INDEL

**13) Filters variant calls using a quality criteria**

GenomeAnalysisTK-1.5-31/GenomeAnalysisTK.jar

-R /disk/Pigref/Pigref.fasta

-T VariantFiltration

--variant /disk/S1/indel_S1.raw.vcf

-o /disk/S1/filtered_indel_S1.vcf

--filterExpression "MQ0 >= 4 && ((MQ0 / (1.0*DP)) > 0.1)"

--filterName "HARD_TO_VALIDATE"

--filterExpression "QUAL < 30" --filterName "QualFilter"

--filterExpression "QD < 5.0" --filterName "QD5"

--filterExpression "FS > 200.0" --filterName "FS200"

GenomeAnalysisTK-1.5-31/GenomeAnalysisTK.jar

-R /disk/Pigref/Pigref.fasta

-T VariantFiltration

--variant /disk/S1/snp_S1.raw.vcf

-o /disk/S1/filtered_snp_S1.vcf

--clusterSize 3 --clusterWindowSize 10

--mask /disk/S1/filtered_indel_S1.vcf --maskName "InDel"

--filterExpression "MQ0 >= 4 && ((MQ0 / (1.0*DP)) > 0.1)"

--filterName "HARD_TO_VALIDATE"

--filterExpression "QUAL < 30" --filterName "QualFilter"

--filterExpression "FS > 200.0 " --filterName "FS200"

**14) Obtain ‘PASS’ field and bi-allele and alternative mono-allele**.

**Literature Cited**

1. Li, H. *et al.* The sequence alignment/map format and SAMtools. *Bioinformatics* **25**, 2078-2079 (2009).

2. McKenna, A. *et al.* The Genome Analysis Toolkit: a MapReduce framework for analyzing next-generation DNA sequencing data. *Genome research* **20**, 1297-1303 (2010).

3. Nielsen, R., Korneliussen, T., Albrechtsen, A., Li, Y. & Wang, J. SNP calling, genotype calling, and sample allele frequency estimation from New-Generation Sequencing data. *PLoS One* **7**, e37558 (2012).
