# Supplementary material for: A genome-wide scan for signatures of directional selection in domesticated pigs
Source: BMC Genomics. 2015 Feb 25;16(1):130. doi: 10.1186/s12864-015-1330-x (PMC4349229; doi:10.1186/s12864-015-1330-x)
Supplement: Additional file 19: Table S6. — Demographic parameter estimation. [file 12864_2015_1330_MOESM19_ESM.docx]

**Supplementary Table S6. Demographic parameter estimation**

| **Parameter*** | **Estimation** | **S.D.** | ***N_Y_*** | 0.834 | 0.211 |
| --- | --- | --- | --- | --- | --- |
| ***N_W_*** | 0.174 | 0.092 | ***m_W-B_*** | 2.872 | 1.116 |
| ***N_B_*** | 0.261 | 0.109 | ***m_W-D_*** | 0.506 | 0.174 |
| ***N_L0_*** | 0.043 | 0.021 | ***m_Y-L_*** | 3.793 | 1.113 |
| ***N_L_*** | 0.425 | 0.186 | ***T_B_*** | 0.667 | 0.343 |
| ***N_Y0_*** | 0.57 | 1.289 | ***T_D_*** | 0.036 | 0.02 |

*Hudson’s *ms* command: '-n 1 0.173766 -n 2 0.425010 -n 3 0.833559 -eg 0 2 126.193496 -eg 0 3 21.012953 -ma x 5.743325 1.011269 5.743325 x 7.586715 1.011269 7.586715 x -ej 0.018089 3 2 -en 0.018089 2 0.260589 -ema 0.018089 3 x 5.743325 x 5.743325 x x x x x -ej 0.351614 2 1
